# A plasma membrane microdomain compartmentalizes ephrin-generated cAMP signals to prune developing retinal axon arbors

**DOI:** 10.1038/ncomms12896

**Published:** 2016-10-03

**Authors:** Stefania Averaimo, Ahlem Assali, Oriol Ros, Sandrine Couvet, Yvrick Zagar, Ioana Genescu, Alexandra Rebsam, Xavier Nicol

**Affiliations:** 1 Sorbonne Universités, UPMC University Paris 06, UMR_S 968, Institut de la Vision, Paris F-75012, France; 2 CNRS, UMR_7210, Paris F-75012, France; 3 INSERM, UMR_S 968, Paris F-75012, France; 4 Sorbonne Universités, UPMC University Paris 06, UMR_S 839, Paris F-75005, France; 5 INSERM UMR_S 839, Paris F-75005, France; 6 Institut du Fer à Moulin, Paris F-75005, France

## Abstract

The development of neuronal circuits is controlled by guidance molecules that are hypothesized to interact with the cholesterol-enriched domains of the plasma membrane termed lipid rafts. Whether such domains enable local intracellular signalling at the submicrometre scale in developing neurons and are required for shaping the nervous system connectivity *in vivo* remains controversial. Here, we report a role for lipid rafts in generating domains of local cAMP signalling in axonal growth cones downstream of ephrin-A repulsive guidance cues. Ephrin-A-dependent retraction of retinal ganglion cell axons involves cAMP signalling restricted to the vicinity of lipid rafts and is independent of cAMP modulation outside of this microdomain. cAMP modulation near lipid rafts controls the pruning of ectopic axonal branches of retinal ganglion cells *in vivo*, a process requiring intact ephrin-A signalling. Together, our findings indicate that lipid rafts structure the subcellular organization of intracellular cAMP signalling shaping axonal arbors during the nervous system development.

## Abstract (web summary)

It is unclear what role cholesterol-enriched domains of the plasma membrane play in mediating the development of neuronal circuits. Here, the authors show that such domains localize ephrin-A-induced cAMP signals, causing the pruning of retinal ganglion cell axons.

## Graphical abstract

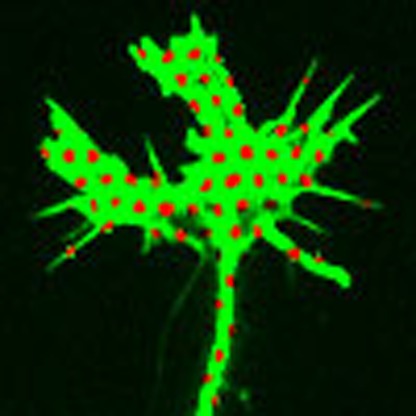

The development of neuronal connectivity requires appropriate signalling downstream of axon guidance molecules to orient axonal growth towards appropriate synaptic partners and to prune ectopic axonal branches present in initially exuberant projection maps^1^. A subset of molecular guidance cues including Netrin-1 and Semaphorin-3A requires lipid rafts to attract or repel axons *in vitro*^2^,^3^. Lipid rafts are highly ordered and dynamic microdomains of the plasma membrane that are enriched in cholesterol, sphingolipids and gangliosides, and are crucial for a wide range of signalling pathways^4^. Since the formulation of the concept of lipid rafts as a membrane compartment with specific protein composition, neurons have provided several examples of raft-dependent signalling pathways *in vitro*^5^ including the modulation of axon turning and outgrowth induced by attractive or repulsive cues^2^,^6^. However, defects in neuronal connectivity or behavioural phenotypes suggesting a general perturbation of axon pathfinding have not been reported in animal models lacking structural components of a subset of lipid rafts including caveolins and flotillins^7^,^8^,^9^,^10^, challenging the potential involvement of lipid rafts in the development of neuronal connectivity.

In cultured cell lines, subtypes of adenylyl cyclases (ACs) are differentially targeted to or excluded from lipid rafts^11^. ACs synthesize the ubiquitous second messenger cyclic AMP (cAMP), and their subcellular localization creates microdomains of this cyclic nucleotide in non-neuronal cells^12^,^13^. cAMP is crucial for axonal response to a large set of guidance molecules including Netrin-1, Semaphorin-3A and ephrin-A5 (refs 14, 15, 16). This ubiquitous second messenger is also involved in a wide range of other cellular processes including neuronal survival, differentiation and migration. Subcellular restriction of cAMP signals might explain how this ubiquitous second messenger achieves specificity for a variety of downstream signalling pathways^17^. In neurons, morphologically-defined compartments of cAMP signalling have been detected *in vitro*, with distinct interactions between cAMP- and calcium-mediated signalling in filopodia and the centre of the growth cones^18^,^19^. However, the biochemical identity of cAMP domains has not been identified.

Although several members of the AC family are expressed in sensory systems, only AC1 appears to be involved in the development of neuronal connectivity^20^. AC1 is crucial for the refinement of sensory axon arbors in the visual and somato-sensory system^21^,^22^. Other ACs expressed in retinal ganglion cells (RGCs) and their targets are dispensable for the development of retinal projections^23^, indicating limited functional redundancy between members of the AC family and suggesting that they generate distinct cAMP signals. However, the spatiotemporal features of the signals involved in the development of neural connectivity are poorly understood. Since AC1 is targeted to lipid rafts in cell lines^24^, this plasma membrane microdomain is a potential candidate to confine cAMP signalling required for the development of neuronal connectivity.

Here, we provide direct evidence that lipid rafts are crucial for the development of RGC axon connectivity *in vivo*, and define microdomains of cAMP signalling in the growth cone of RGCs. We found that ephrin-As, a set of axon guidance molecules, required for the development of retinal topography^25^, induce a lipid raft-specific reduction in cAMP concentration in RGC growth cones. Lipid raft-specific cAMP signals are required for both ephrin-A-induced axon retraction *in vitro* and pruning of RGC arbors in the superior colliculus (SC) *in vivo*. These results indicate that lipid raft-segregated cAMP signals are functionally relevant in developing neurons and are required for shaping the precise connectivity of the nervous system.

## Results

### Ephrin-A-induced axonal retraction requires lipid rafts

**Figure 1:**
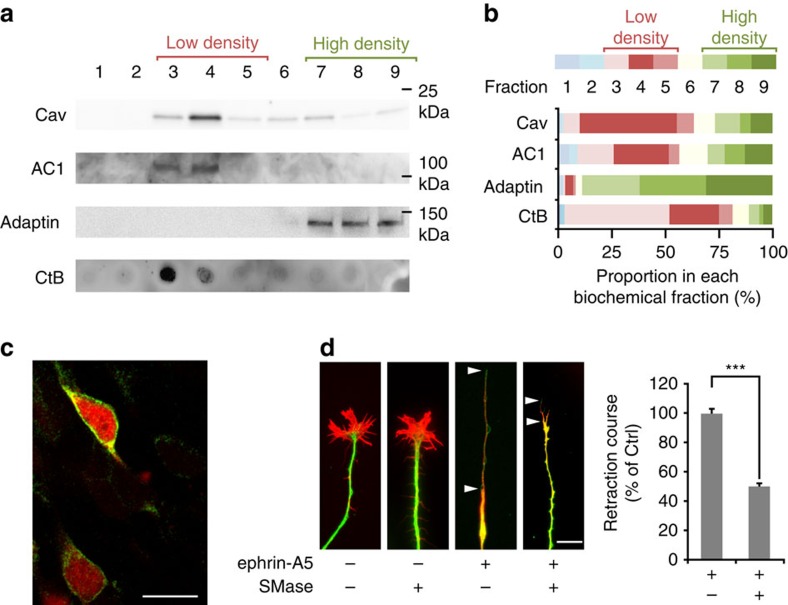
Lipid rafts contain AC1 and are required for ephrin-A-induced axonal retraction. (**a**) AC1 fused to GFP and overexpressed in the developing retina is detected in fractions 3 and 4 after sucrose-density gradient fractionation of the plasma membrane. This coincides with the location of the lipid raft markers Caveolin-1 (Cav, enriched in fractions 3 and 4) and cholera toxin (CtB), a lipid raft marker that binds ganglioside M1, other gangliosides, and raft-targeted glycoproteins (enriched in fractions 3 and 4). AC1 is excluded from the fractions enriched in β-Adaptin (7–9), a marker of the non-raft fraction of the membrane. (**b**) Proportion of Caveolin, AC1, β-Adaptin and CtB expression found in each biochemical fraction. For each marker detected, the optical density (OD) of the bands in each fraction is quantified and normalized to the sum of the OD in all fractions. The proportion of the signal found in each fraction is shown. Each biochemical fraction is colour-coded. Red tones code for the low-density Caveolin- and CtB-enriched fractions (3–5), whereas green tones denote the high-density β-Adaptin-enriched fractions (7–9). Cav, Adaptin, AC1, CtB *n*=3 independent experiments. (**c**) Overexpressing AC1 fused to GFP (green) in retinal neurons co-electroporated with mCherry (cytoplasmic localization, red) does not affect AC1 plasma membrane targeting. Scale bar, 10 μm. *n*=3 experiments from independent cultures. (**d**) Altering lipid rafts integrity with SMase does not affect the morphology of growing RGC axons. Ephrin-A5 induces a collapse of RGC growth cones *in vitro* and a subsequent axonal retraction leaving a long trailing process (encompassed by the two arrowheads). SMase does not affect the collapse of the growth cone but reduces axon retraction measured as the length of the trailing process (between the two arrowheads). Scale bar, 10 μm. *n*≥360 axons per condition (ctrl *n*=600, SMase *n*=360) from three independent cultures. Data are mean±s.e.m. ***P≤0.001, Mann–Whitney test. Uncropped gels are provided in Supplementary Fig. 8.

To evaluate whether lipid rafts define a potential compartment of RGCs from which cAMP signalling originates, we analyzed the subcellular targeting of AC1, the AC required for the development of retinal projections. We identified the subcellular localization of AC1 in mouse E13.5 retina electroporated using a green fluorescent protein (GFP)-tagged version of AC1. GFP fusion did not affect the targeting of AC1 to the plasma membrane (Fig. 1c). Sucrose-density gradient fractionation was used to biochemically isolate high- and low-density fractions of the plasma membrane prepared from retina. AC1 was found highly enriched in low-density fractions labelled by Caveolin-1, a protein of lipid rafts, and by Cholera toxin β subunit (CtB), a marker binding gangliosides^26^ and glycoproteins^27^, which are both components of lipid rafts (Fig. 1a,b). This suggests that this subcellular compartment contains the AC1-dependent cAMP signals that regulate ephrin-A-induced axonal retraction and RGC axon connectivity. To determine whether lipid rafts are required for axonal response to ephrin-As, cultured retinal explants in which lipid raft integrity was altered using sphingomyelinase (SMase) were exposed to ephrin-A5. SMase specifically hydrolyzes sphingomyelins, one of the main components of lipid rafts together with cholesterol, resulting in the removal of cholesterol and associated proteins from the plasma membrane^28^. Although the amount of sphingomyelin degraded by SMase was not assessed, we verified that treating retinal explants with SMase prevented the enrichment of the lipid raft marker CtB in low-density fractions (Supplementary Fig. 1). The general reduction of CtB labelling (Supplementary Fig. 1) was expected since SMase treatment results in the removal of lipid rafts components from the plasma membrane^28^. These observations confirm that SMase modifies the structure of the lipid rafts. SMase alone did not perturb the morphology of RGC growth cones (Fig. 1d). On ephrin-A application, untreated axons collapsed and retracted leaving a long single trailing process. In contrast, SMase-treated axons collapsed but the length of the retraction process was reduced (Fig. 1d), mimicking the phenotype of *AC1*^*−/−*^ axons^15^. SMase treatment produces ceramide, a lipid that might in turn activate signalling pathways potentially interfering with axon retraction. To rule out this possibility, we perturbed lipid raft integrity by cholesterol oxidation with cholesterol oxidase (COx), a treatment that does not produce ceramide. COx drastically reduced the enrichment of CtB in low-density fractions prepared from retinal explants, confirming that this treatment disrupts the structure of lipid rafts (Supplementary Fig. 1). COx-treated axons collapsed when exposed to ephrin-A5, but the length of their retraction process was reduced, mimicking the effect of SMase (Supplementary Fig. 2). This indicates that SMase metabolites, including ceramide, are not responsible for the reduced retraction process. This was confirmed by exposing retinal axons to ceramide before ephrin-A5-induced axonal retraction. This treatment did not affect the length of the trailing process observed after retraction (Supplementary Fig. 2). These observations demonstrate that lipid rafts contain AC1, the cAMP synthesizing enzyme required for ephrin-A5-induced repulsion of RGC growth cones, and are involved in axon retraction in response to this axon guidance molecule.

### Ephrin-A5 induces a reduction in cAMP near lipid rafts

**Figure 2:**
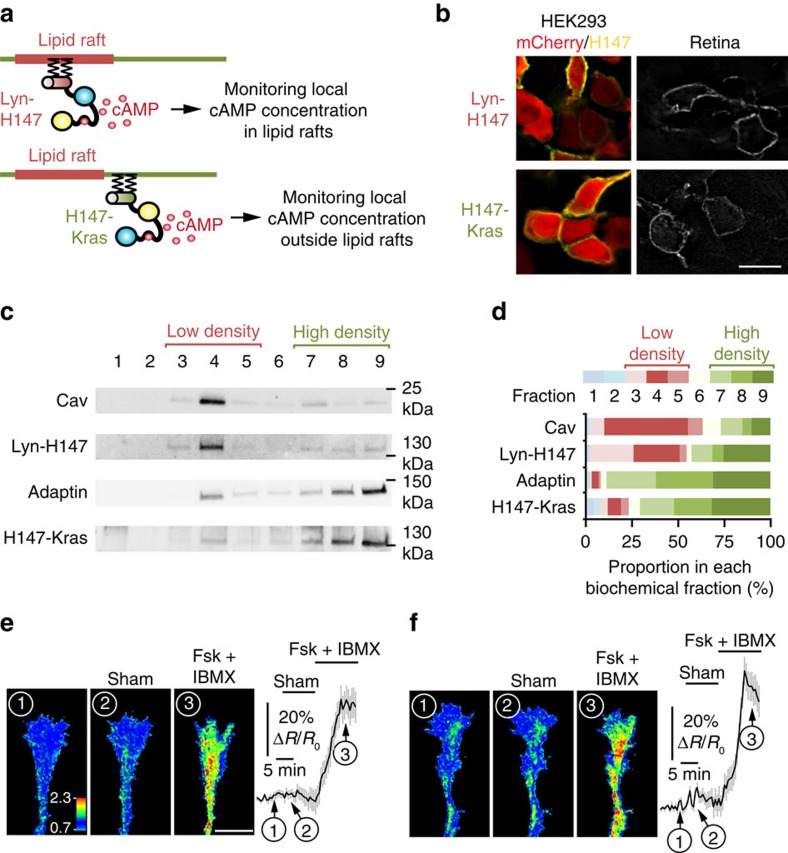
Monitoring local cAMP inside or outside the submembrane domain adjacent to lipid rafts. The strategy used to monitor cAMP signals in the vicinity of the raft or non-raft domains of the plasma membrane is schematized in **a**. The cAMP-sensitive FRET sensor H147 is fused to either a tandem of two Lyn sequences in 5′ or to a Kras sequence in 3′ to confine the probe inside or outside lipid rafts, respectively and monitor local cAMP signals. (**b**) Electroporation of Lyn-H147 or H147-Kras in retinal explants (right panels) or co-transfection of each FRET probe (yellow) with mCherry (red) in HEK293 cells (left panels) leads to a plasma membrane-restricted expression of both sensors. Scale bar, 10 μm. *n*=3 independent cultures (HEK293) or three retinas. (**c**) Lyn-H147 is enriched in the biochemically-isolated fractions enriched in the lipid raft marker Caveolin-1 (fractions 3 and 4), whereas H147-Kras is mostly found in the fractions enriched in the lipid raft-excluded marker β-Adaptin (fractions 7–9). (**d**) Proportion of Caveolin, Lyn-H147, β-Adaptin and H147-Kras expression found in each biochemical fraction. Each biochemical fraction is colour-coded. Red tones code for the low-density fractions (3–5), whereas green tones denote the high-density fractions (7–9). Cav, Adaptin, Lyn-H147, H147-Kras *n*=3 independent experiments. (**e**) Pharmacological increase of cAMP after Fsk and IBMX exposure leads to an increase of the CFP/FRET ratio detected by the Lyn-H147 sensor (lipid raft-targeted) in RGC growth cones. The CFP/FRET ratio is not affected by sham stimulation (*n*=14 growth cones from four independent cultures). The CFP/FRET ratio is colour-coded from blue (low ratio, low cAMP concentration) to red (high ratio, high cAMP concentration). (**f**) Fsk and IBMX induce an increase of the CFP/FRET ratio of the lipid raft-excluded cAMP sensor H147-Kras, whereas sham stimulation does not (*n*=9 growth cones from three independent cultures). CFP/FRET ratio is coded as in **e**. Data are mean±s.e.m. (**e**,**f**) Scale bar, 10 μm. Uncropped gels are provided in Supplementary Fig. 8.

To evaluate whether lipid rafts compartmentalize cAMP signals in axonal growth cones, we monitored cAMP concentration in and outside lipid raft submembrane domain. An existing cAMP FRET sensor, H147 (ref. 29), was targeted to each compartment to analyze local cAMP modulation. Targeting lipid rafts was achieved using the 5′ insertion of two palmitoylation–myristoylation tandems derived from Lyn kinase^13^ (Fig. 2a). H147 was targeted to the plasma membrane and excluded from lipid rafts by a 3′ fusion to a CaaX sequence along with a polylysine motif derived from K-Ras^13^ (Fig. 2a). The lipid raft-targeted (Lyn-H147) and excluded (H147-Kras) sensors were found at the plasma membrane in transfected HEK293 cells and in electroporated retinas (Fig. 2b), and their specific subcellular localization was validated using membrane fractionation with a sucrose-density gradient. Lyn-H147 was found in the same membrane fractions as Caveolin-1 (Fig. 2c,d). In contrast, H147-Kras was highly enriched in the biochemical fractions of the plasma membrane containing the lipid raft-excluded protein β-Adaptin (Fig. 2c,d). Expression of either Lyn-H147 or H147-Kras did not affect growth cone morphology (Supplementary Fig. 3). Both sensors were able to detect cAMP variations in axonal growth cones after exposure to the AC activator Forskolin (Fsk) combined with the non-specific phosphodiesterase inhibitor IBMX. The computed CFP/FRET ratio, reflecting the cAMP concentration, did not change significantly after sham stimulation (Fig. 2e,f).

**Figure 3:**
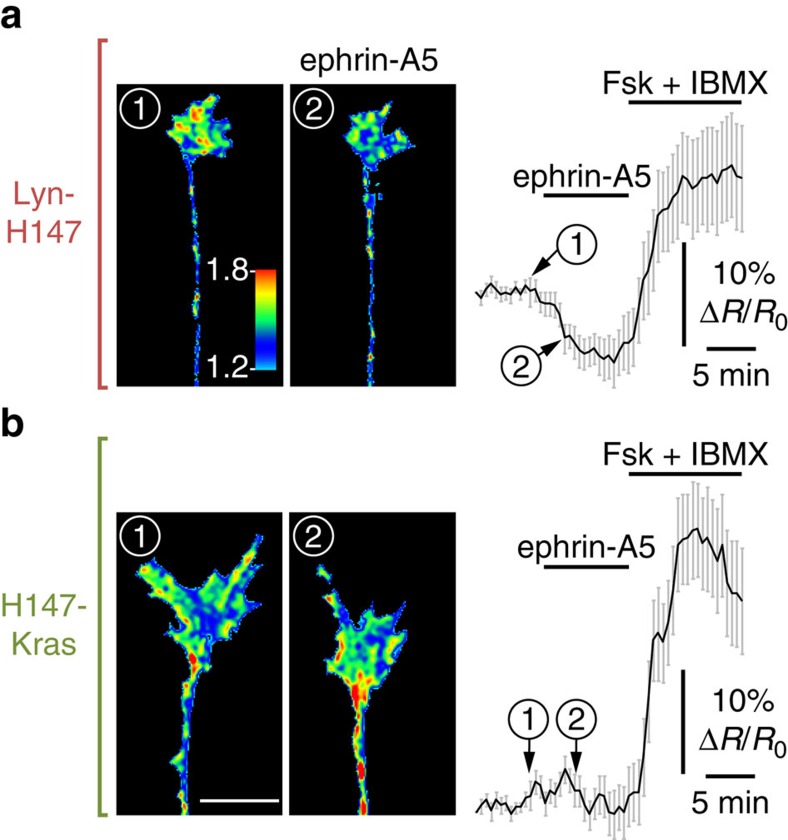
Ephrin-A5 induces a reduction in cAMP concentration restricted to the vicinity of lipid rafts. The computed CFP/FRET ratio is shown in RGC axons expressing either the lipid raft-targeted cAMP sensor Lyn-H147 or the raft-excluded probe H147-Kras, revealing the local variation in cAMP concentration. The ratio is colour-coded from blue (low ratio, low cAMP concentration) to red (high ratio, high cAMP concentration). Axon viability is controlled at the end of the experiment, verifying that the cAMP level increases on stimulation of the transmembrane ACs with Forskolin (Fsk), together with the phosphodiesterases inhibitor IBMX. (**a**) Ephrin-A5 induces a reduction in cAMP concentration in the vicinity of lipid rafts (colour of the growth cone changing from green/red into blue/green), whereas (**b**) the CFP/FRET ratio of the biosensor excluded from lipid rafts is unaffected by ephrin-A5. Scale bar, 10 μm. (**a**) *n*=25 axons from seven independent cultures. (**b**) *n*=19 axons from five independent cultures. Data are mean±s.e.m.

These subcellularly targeted FRET sensors were electroporated in retinal explants, and cAMP concentration was monitored in growth cones exposed to ephrin-A5. This repellent guidance cue induced a reduction in the CFP/FRET ratio of Lyn-H147-expressing growth cones (Fig. 3a), revealing a decrease in cAMP near lipid rafts. This reduction in cAMP concentration was absent after sham stimulation (Fig. 2e). cAMP concentration reached a plateau 8 min after stimulation (Fig. 3a). In contrast, the cAMP concentration monitored by H147-Kras next to the non-raft fraction of the plasma membrane was not affected by ephrin-A5 (Fig. 3b). This indicates that ephrin-A5 modulates cAMP concentration specifically in the submembrane region adjacent to lipid rafts and does not affect the concentration of this cyclic nucleotide next to other membrane compartments.

### Axonal retraction requires cAMP signalling near lipid rafts

**Figure 4:**
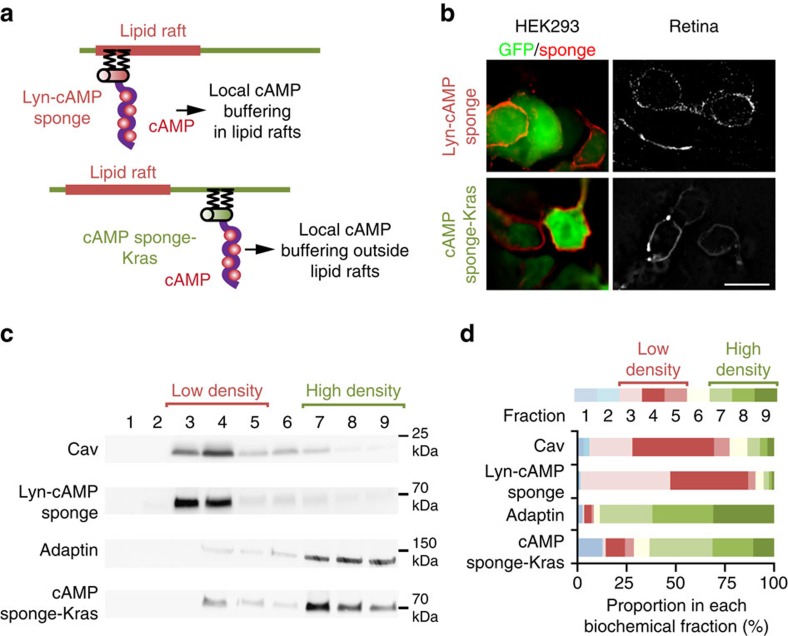
Lipid raft targeting or exclusion of a cAMP signalling blocker. The strategy used to block raft and non-raft cAMP signals is schematized in **a**. The genetically-encoded cAMP blocker ‘cAMP sponge' is fused to either a tandem of two Lyn sequences in 5′ or a Kras sequence in 3′ to target the blocker to lipid rafts or exclude it from this plasma membrane compartment, respectively. (**b**) Lyn-cAMP sponge and cAMP sponge-Kras are both restricted to the plasma membrane in developing retinal neurons (right panels) or when cotransfected with GFP (green) in HEK293 cells (left panels). Scale bar, 10 μm. *n*=3 independent cultures (HEK293) or three retina. (**c**) Lyn-H147 is enriched in the biochemically-isolated fractions enriched in the lipid raft marker Caveolin (3 and 4), whereas H147-Kras is mostly found in the compartment enriched in the non-raft marker β-Adaptin (fractions 7–9). (**d**) The proportion of the signal found in each biochemical fraction is shown. The identity of each fraction is colour-coded. Red tones code for low-density fractions (3–5), whereas green tones denote high-density fractions (7–9). Cav, Adaptin, Lyn-cAMP sponge, cAMP sponge-Kras *n*=3 independent experiments. Uncropped gels are provided in Supplementary Fig. 8.

The existence of distinct local cAMP signals suggested a differential role of cAMP signalling in the submembrane space proximal and distal from lipid rafts during ephrin-A-stimulated axon retraction. To further investigate the cellular signalling dependent on lipid rafts, a genetically-encoded chelator of cAMP termed ‘cAMP sponge'^30^ was targeted to or outside lipid rafts, enabling local perturbation of cAMP downstream signalling. cAMP sponge subcellular localization was performed using the same targeting sequences as for the FRET sensor H147: tandem of two Lyn sequences in 5′ for lipid rafts and a Kras sequence for the non-raft compartment (Fig. 4a). Both local cAMP signalling inhibitors were targeted to the plasma membrane (Fig. 4b). Appropriate subcellular localization of Lyn-cAMP sponge and cAMP sponge-Kras was verified biochemically. Lyn-cAMP sponge was highly enriched in the same membrane fractions as the lipid raft marker Caveolin-1, whereas cAMP sponge-Kras was mainly excluded from this compartment and was detected in fractions containing the non-raft marker β-Adaptin (Fig. 4c,d). Lyn-cAMP sponge and Lyn-H147 or cAMP sponge-Kras and H147-Kras were co-electroporated in retinal explants. The expression of each targeted cAMP sponge was sufficient to reduce the increase of cAMP concentration induced by Fsk and IBMX confirming their ability to interfere with cAMP signalling (Supplementary Fig. 4).

**Figure 5:**
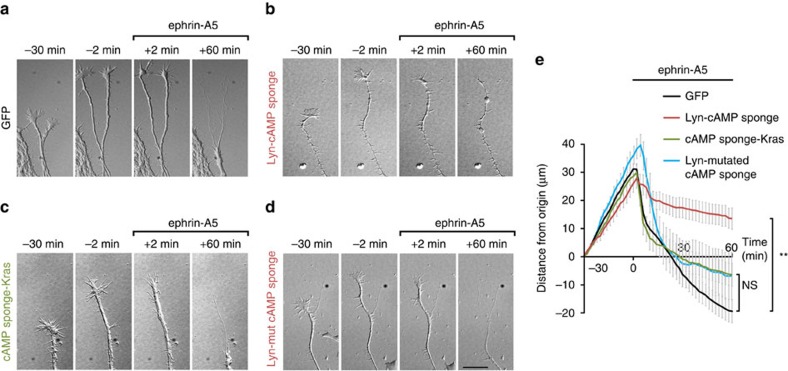
cAMP signalling is required within but not outside the submembrane domain of lipid rafts for ephrin-A5-induced retraction of RGC axons. (**a**) GFP-expressing axons growing before ephrin-A5 addition to the culture medium (−30 min to −2 min), collapse shortly after ephrin-A5 application (+2 min) and retract during 60 min after ephrin-A5 exposure. (**b**) In contrast, axons expressing Lyn-cAMP sponge, in which cAMP signalling is blocked specifically in the vicinity of lipid rafts, the retraction is drastically reduced following the initial collapse. Axons expressing Lyn-cAMP sponge are identified by co-electroporated GFP (Supplementary Fig. 5). Lyn-cAMP sponge does not perturb axon outgrowth before ephrin-A5 application. (**c**) The blockade of cAMP near the plasma membrane but away from lipid rafts or (**d**) the expression of a variant of Lyn-cAMP sponge unable to bind cAMP does not affect ephrin-A5-induced axon retraction. (**e**) The distance of the growth cone from its initial position (*t*=−30 min) is plotted. Axons are exposed to ephrin-A5 at *t*=0 min. GFP-, cAMP sponge-Kras- and Lyn-cAMP mutated sponge-expressing axons grow and retract similarly, whereas the ephrin-A5-induced retraction of Lyn-cAMP sponge-expressing axons is reduced. Scale bar, 15 μm. (**a**) *n*=76 axons from 13 cultures. (**b**) *n*=19 axons from 7 cultures. (**c**) *n*=22 axons from 5 cultures. (**d**) *n*=15 axons from 4 cultures. Data are mean±s.e.m. **P≤0.01, Kruskal–Wallis test.

Both Lyn-cAMP sponge and cAMP sponge-Kras were fused to a red fluorescent protein (mCherry and mRFP, respectively). However, limited brightness prevented the direct identification of Lyn-cAMP sponge in living RGC axons. To overcome this limitation, GFP was co-electroporated with an excess of either Lyn-cAMP sponge or cAMP sponge-Kras in E13.5 retinas *ex vivo*. All GFP-expressing axons were positive for Lyn-cAMP sponge after mCherry-immunostaining (Supplementary Fig. 5). Growing axons expressing either the raft-targeted or the raft-excluded blocker of cAMP signalling were monitored during exposure to ephrin-A5. Axons with unaffected cAMP signalling collapsed and retracted after superfusion of ephrin-A5-containing medium (Fig. 5a; Supplementary Movie 1). cAMP buffering near lipid rafts by Lyn-cAMP sponge did not affect growth cone collapse, but subsequent axon retraction was slowed (Fig. 5b,e; Supplementary Movie 1), mimicking the phenotype of *AC1*^*−/−*^ RGCs^15^. In contrast, the blockade of cAMP signalling next to the non-raft fraction of the plasma membrane had no effect on the retraction rate of RGC axons exposed to ephrin-A5 (Fig. 5c,e; Supplementary Movie 1). Retracting axons expressing a variant of Lyn-cAMP sponge unable to bind and buffer cAMP^30^ were not distinguishable from GFP-expressing axons (Fig. 5d,e; Supplementary Movie 1). Axon outgrowth before ephrin-A exposure was not affected by either local cAMP blockade (Fig. 5; Supplementary Movie 1). These results demonstrate that local cAMP signalling in the vicinity of lipid rafts but not outside this submembrane region is required for ephrin-A-induced axonal retraction *in vitro.*

### cAMP signals near lipid rafts suffice for axon retraction

**Figure 6:**
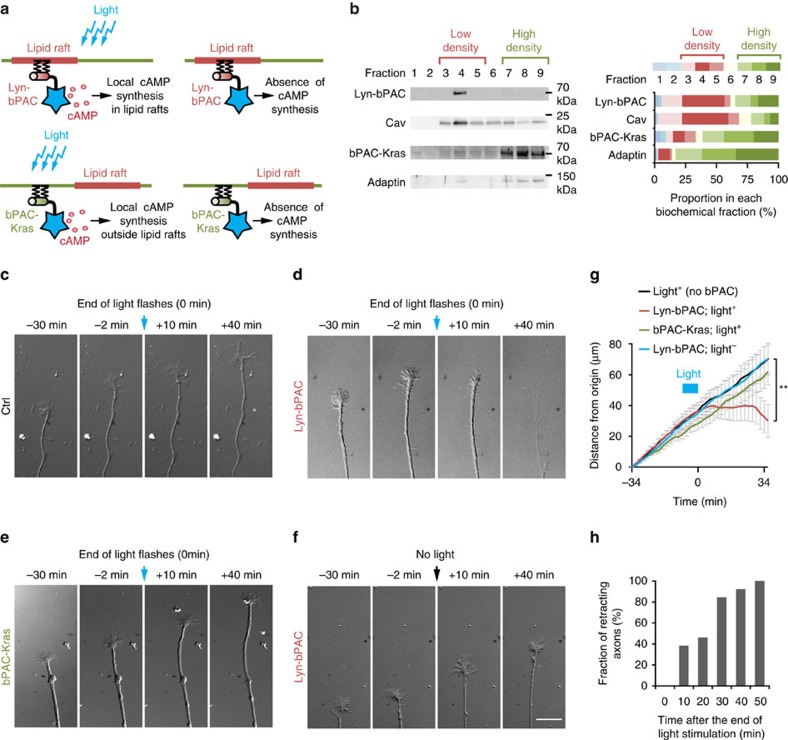
A cAMP decrease in the vicinity of lipid rafts is sufficient to induce retraction of RGC axons. The strategy used to manipulate cAMP signals with subcellular resolution is schematized in **a**. The light-sensitive AC bPAC is fused to either a tandem of two Lyn sequences in 5′ or a Kras sequence in 3′ to target the blocker to lipid rafts or exclude it from this plasma membrane compartment, respectively. Blue light induces a local increase of cAMP followed by a reduction of its concentration after the release of light stimulation (Supplementary Fig. 6). (**b**) Lyn-bPAC is highly enriched in fractions 3–5 together with the lipid raft marker Caveolin. The proportions of Lyn-bPAC and Caveolin-1 in each biochemical fraction are shown. Each biochemical fraction is colour-coded. Red tones code for the low-density fractions (3–5), whereas green tones denote the high-density β-Adaptin-enriched fractions (7–9). Cav *n*=6 independent experiments; Adaptin, Lyn-bPAC, bPAC-Kras *n*=3 independent experiments. Blue light does not affect outgrowth of (**c**) wild-type or (**e**) bPAC-Kras-expressing axons. (**d**) Light exposure (starting at *t*=0 min) induces retraction of Lyn-bPAC-expressing axons after light stimulation was turned off. (**f**) Outgrowth of Lyn-bPAC-expressing axons is not affected without blue light illumination. (**g**) The distance of the growth cone from its initial position (*t*=−34 min) is plotted. Light activation of bPAC is interrupted at *t*=0 min. Control, Lyn-bPAC- and bPAC-Kras-expressing axons grow similarly and are not affected by the onset of light exposure. Interrupting light exposure induces stalling or retraction in Lyn-bPAC-expressing axons but not in control or bPAC-Kras-expressing axons. No bPAC/Light^+^ *n*=15 from four independent cultures; Lyn-bPAC/Light^+^ *n*=14 from five independent cultures; bPAC-Kras/Light^+^ *n*=8 from three independent cultures; Lyn-bPAC/Light^+^ *n*=14 from four independent cultures. (**h**) Lyn-bPAC-expressing axons retract after but not during light exposure. The variability of the retraction onset reflects the variability of the timing of cAMP decrease (Supplementary Fig. 6e). Scale bar, 15 μm. Data are mean±s.e.m. **P≤0.01, Kruskal–Wallis test.

Since ephrin-A5 elicits a reduction in cAMP concentration in the vicinity of lipid rafts, we investigated whether a cAMP reduction restricted to this subcellular domain is sufficient to induce axon retraction. To examine the effects of a drop in cAMP, we controlled cAMP synthesis using the light-sensitive AC bPAC^31^. cAMP level was artificially elevated by a sustained light activation of bPAC. Removing light activation causes a reduction of cAMP resulting from the activity of phosphodiesterases. This biphasic phosphodiesterase-dependent response has been reported using bPAC and a similar photoactivatable AC in *Xenopus* oocytes, HEK293 cells and hippocampal neurons^31^,^32^. bPAC was tagged with mRFP and targeted to or excluded from lipid rafts with a tandem of two Lyn sequences (Lyn-bPAC) or a Kras motif (bPAC-Kras), respectively (Fig. 6a). The subcellular restriction of Lyn-bPAC and bPAC-Kras was confirmed using plasma membrane fractionation (Fig. 6b), and targeted bPACs were electroporated in embryonic retinas *ex vivo*. Overall, 1–5 flashes of 470 nm light were sufficient to induce an increase of cAMP concentration followed by the hydrolysis of this cyclic nucleotide, reproducing the previously reported biphasic response (Supplementary Fig. 6). RGC axons expressing either Lyn-bPAC or bPAC-Kras and exposed to 1–5 flashes of 470/40 nm light were monitored. Axons were unaffected by the onset of light activation (Fig. 6c–h). Interrupting light activation of Lyn-bPAC induced axon stalling or retraction in 77% of the cases, whereas Lyn-bPAC-expressing axons were unaffected in absence of light exposure (Fig. 6d,f,g; Supplementary Movie 2). All light-responsive Lyn-bPAC-expressing axons stalled or retracted later than the end of the light stimulus (Fig. 6h), suggesting that axon retraction is induced by the reduction in cAMP concentration and not by the increase of the concentration following the onset of Lyn-bPAC stimulation. The onset of cAMP reduction following the release of Lyn-bPAC stimulation was variable (Supplementary Fig. 6e), reflecting the variability of the onset of axon retraction. Non-electroporated axons and axons expressing bPAC-Kras were unaffected by the release of light exposure (Fig. 6c,e,g; Supplementary Movie 2). Taken together, these experiments suggest that a dynamic and lipid raft-restricted reduction in cAMP concentration is sufficient to mimic the ephrin-A-induced retraction of RGC axons. This confirms previous reports describing the requirement of a dynamic cAMP regulation for axon pathfinding^18^,^33^.

### RGC axon pruning requires cAMP signalling near lipid rafts

During the development of retinal projections, retinal axons initially overshoot their final termination zone and extend in the most caudal part of the SC. The subsequent refinement of axonal arbors and the retraction of their caudal branches is dependent on ephrin-As^25^. To evaluate whether the local cAMP signals required for ephrin-A *in vitro* are involved in shaping retinal arbors *in vivo*, we investigated the impact of local cAMP signalling perturbation in the submembrane domain of lipid rafts during the development of retinal projections in the SC. Lyn-cAMP sponge or cAMP sponge-Kras were co-electroporated with GFP in E15.5 retinas *in utero*, and the SC and retinas of electroporated animals were harvested at P10. Individual RGC axon arbors in the SC were imaged using a confocal microscope and manually reconstructed (Supplementary Movie 3). E15.5 electroporation enables sparse expression of the constructs of interest enabling tracking and reconstruction of single RGC axons in the SC. Electroporated RGCs reside in the centre of the retina and their projections were largely located in the middle of the SC along the rostro-caudal axis.

**Figure 7:**
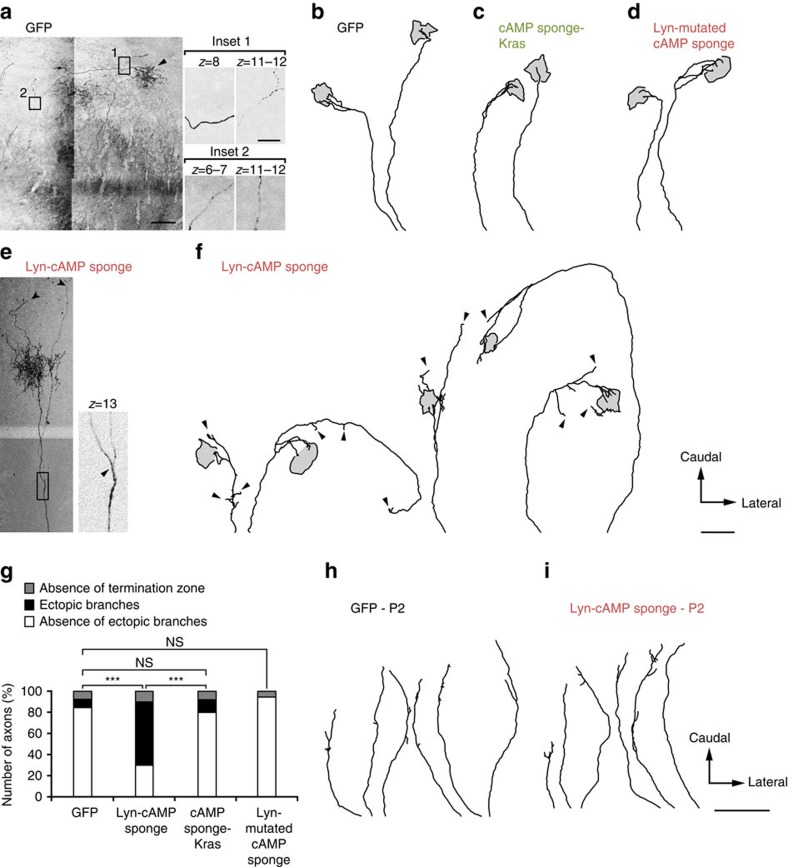
cAMP signalling inside but not outside the submembrane domain of lipid rafts is required for RGC axon arbor refinement in the SC. *In utero* retinal electroporation of (**a**,**b**) GFP, (**c**) cAMP sponge-Kras, (**d**) the mutated version of Lyn-cAMP unable to bind cAMP or (**e**,**f**) Lyn-cAMP sponge. Examples of reconstruction of electroporated RGC arbors at P10 in the SC are shown for each condition. The extent of the terminal arborization (arrowhead in **a**) is identified as a grey area delineated with a black contour. The rostral limit of the SC corresponds to the bottom of each trace. GFP-electroporated axons exhibit a dense terminal zone and an absence of branch tip outside the termination zone. In contrast, exuberant branches are detected in Lyn-cAMP sponge-expressing axons (arrowheads in **e**,**f**). These branches do not terminate in the dense termination zone. cAMP sponge-Kras and Lyn-mutated cAMP sponge-expressing axons were not distinguishable from GFP-expressing arbors, with an absence of branch tips outside of the dense termination zone. The insets in **a**,**e** highlight distinct confocal sections of the image and enable the distinction between axon crossing (**a**) and branching (**e**). (**g**) Number of axons without a termination zone and with or without ectopic branches. GFP, *n*=26 from nine animals; Lyn-cAMP sponge, *n*=30 from eight animals; cAMP sponge-Kras, *n*=25 from three animals; Lyn-mutated cAMP sponge, *n*=22 from three animals. ***P≤0.001, *χ*^2^ test. Reconstruction of (**h**) GFP or (**i**) Lyn-cAMP sponge-electroporated axons at P2. In both cases axons were poorly branched. Scale bar in **a** 100 μm, in inset 20 μm; applies for **e**. Scale bar in **f** 300 μm; applies for **b**–**d**. Scale bar in **i** 300 μm; applies for **h**.

Axons of GFP-electroporated RGCs are generally refined: they retract from the caudal SC to target a focal termination zone in the middle of the rostro-caudal axis. They elaborate a dense terminal arbor without ectopic branches along the axon shaft (Fig. 7a,b,g). Projections co-expressing GFP and Lyn-cAMP sponge exhibit a dense terminal arbor but fail to eliminate ectopic branches. The remaining exuberant branches are located along the shaft of the axon and branches could be found caudally to the termination zone (Fig. 7e–g). This defect is reminiscent of the phenotype of AC1-deficient RGC axon arbors *ex vivo* and *in vivo*^15^,^34^. In contrast, altering cAMP signalling in the vicinity of the non-raft fraction of the plasma membrane (with cAMP sponge-Kras) or using a variant of cAMP sponge unable to bind cAMP (Lyn-mutated cAMP sponge) targeted to lipid rafts did not affect the development of RGC axon arbors in the SC (Fig. 7c,d,g). In both cases the terminal arborization developed normally and no ectopic branches were detected. The absence of exuberant arbors in Lyn-mutated cAMP sponge-electroporated axons indicates that overexpression of a lipid raft-targeted protein without cAMP buffering capacity is not sufficient to alter the development of RGC axon arborizations. This demonstrates that cAMP signalling in the vicinity of lipid rafts but not outside this submembrane compartment is required for the refinement of retinal projections *in vivo*.

Since cAMP is an important regulator of RGC axon outgrowth^35^, we examined whether Lyn-cAMP sponge expression affects the retino-collicular pathway before the refinement of the projections. Lyn-cAMP sponge and GFP-electroporated RGC axons were not distinguishable in the SC at P2 before the elimination of ectopic branches. In both conditions, immature RGC axons extended to the caudal end of the SC (Fig. 7h,i), suggesting that axon outgrowth and early development of the retinal pathway are not affected. Taken together our data establish the functional specificity of distinct subcellular cAMP compartments during the development of neuronal connectivity and demonstrate that lipid raft-restricted signals are required for the refinement of axonal wiring *in vivo*.

## Discussion

Our experiments identify the cAMP signals that regulate RGC growth cone response to the ephrin-A repellents. We demonstrate that a local reduction in cAMP concentration restricted to the vicinity of lipid rafts is required for the RGC axon retraction *in vitro* and for axonal arbor refinement *in vivo*. This describes the biochemical identity of submicrometre-scale compartments of cAMP signalling, that may match previously described and morphologically-defined subcellular structures with distinct cAMP signals in developing neurons^18^,^19^. Lipid rafts are involved in axon turning dependent on other guidance molecules including netrin-1 and semaphorin-3A^2^,^3^ *in vitro*, and ephrin-A reverse signalling induces a local Src-dependent phosphorylation restricted to this microdomain^36^,^37^. We extend the role of lipid rafts in axon guidance to ephrin-A forward signalling. This is consistent with the differential structure of EphAs in and outside lipid rafts. Oligomerization of EphAs is required to trigger ephrin-A downstream signalling and occurs preferentially in lipid rafts^38^ where AC1 and the cAMP signals required for axon retraction are located.

In the mature nervous system, lipid rafts are crucial for the regeneration of injured axons *in vivo*^39^,^40^. In contrast, alteration in the development of neuronal connectivity or behavioural phenotypes suggesting a general perturbation of axon pathfinding have not been reported in animal models lacking protein scaffolds required for lipid raft formation including caveolins and flotillins^7^,^8^,^9^,^10^. This is surprising given that the integrity of the lipid raft compartment is required for the response of axons to a subset of guidance molecules *in vitro*^2^,^3^. We provide evidence that altering cAMP signalling specifically in the vicinity of lipid rafts *in vivo* prevents the refinement of RGC axon arbors, identifying a role of this membrane compartment during the development of the nervous system and reconciling *in vitro* data with *in vivo* observations. Future studies might reveal subtle connectivity defects in caveolin or flotillin-deficient mice. These animal models may also have an unaffected neural connectivity because of the heterogeneity of lipid rafts that do not all require a caveolin or flotillin scaffold^41^. The lipid raft subtypes required for axon guidance may not be dependent on these two families of scaffolding proteins.

**Figure 8:**
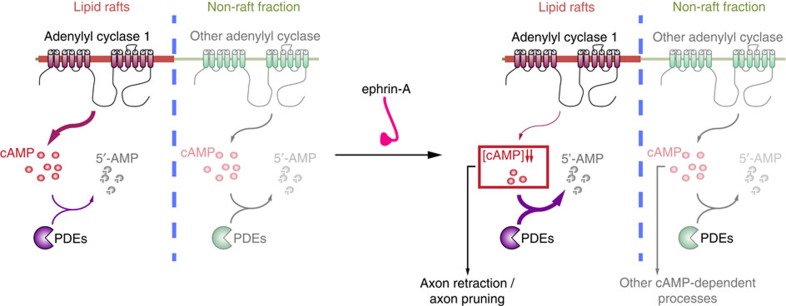
Model of local cAMP signalling for specific activation of downstream signalling. cAMP signals can be generated both in the vicinity and further away from lipid rafts. Near lipid rafts, ephrin-A5 induces a decrease in the cAMP concentration, likely by modifying the balance between local AC and phosphodiesterase activity, without affecting cAMP signals in distal (non-raft) compartments. Lipid raft-proximal signals are required for axon retraction *in vitro* and retinal axon pruning *in vivo*, in contrast to cAMP signal excluded from this domain that might regulate other cellular processes modulated by this second messenger.

Since cAMP is a second messenger involved in the regulation of a wide range of axon guidance molecules^14^,^42^,^43^,^44^, its local signalling in the vicinity of lipid rafts may reflect a specific spatiotemporal signature for the regulation of axon outgrowth directionality. Spatially restricted cAMP modulation is likely to contribute to the specificity of signals regulating axon guidance, limiting cross-talk with the wide range of other cAMP-regulated cellular processes (Fig. 8). Temporal features of cAMP are also crucial for the regulation of axon pathfinding by cAMP^18^,^33^,^45^,^46^. Imposing a sustained low cAMP concentration leads to the reduction of ephrin-A-induced axon retraction *in vitro* and ectopic axonal branches in the SC *in vivo*^15^,^21^,^34^. However, a sustained increase of cAMP also induces a similar retraction phenotype and an exuberant arborization of RGC axons^33^, suggesting that the concentration of cAMP does not determine axonal behaviour. In contrast, pulses, rather than sustained cAMP elevation rescue the retraction of RGC axons when it is blocked by the lack of electrical activity^33^. This rescue is not observed when successive steps of increase in cAMP concentration are imposed together with the blockade of phosphodiesterases, preventing cAMP degradation^33^. This suggests that a dynamic modulation of cAMP concentration is required for growth cones to retract when exposed to ephrin-As. The data presented here confirm this model since both ephrin-A5 and the release of bPAC activation by light induces a single step of cAMP reduction. Ephrin-A5 induces a drop in cAMP in growth cones with an unaffected resting concentration of this second messenger. Light-induced activation of bPAC leads to an initial increase of cAMP concentration followed by a drop in cAMP after the end of light exposure. The initial increase in cAMP does not affect axon outgrowth, whereas axons stall or retract after the end of bPAC activation. This is consistent with axon retraction being induced by a drop in cAMP concentration, independently of the resting cAMP level. This is in agreement with the observation that cAMP sponge prevents axon retraction. cAMP sponge might steadily reduce the resting concentration of cAMP. This bottom cAMP level cannot further decrease when axons are exposed to ephrin-A5 and axon retraction is blocked (Supplementary Fig. 7). The signalling pathway leading to the modulation of cAMP concentration after EphA activation is still unclear. This might include the focal adhesion kinase (FAK)/Src pathway. Indeed, these proteins are crucial for retinal axon response to ephrin-As^47^, and Src family kinases are recruited to EphAs after ephrin-A stimulation^48^. *In vitro* analysis suggests that Src is able to regulate AC activity through the modulation of both Gα_i_ and Gα_s_ subunits of G proteins^49^. This pathway might be responsible for cAMP modulation by ephrin-As, but further experiments will be needed to assess this potential link.

Local cAMP signals are likely to be the result of restricted targeting of ACs^50^. In cell lines, transmembrane ACs are sorted in two subcellular compartments based on their modulation by calcium: calcium-regulated ACs (AC1, 3, 5, 6 and 8) are targeted to lipid rafts, whereas the calcium insensitive ACs (AC2, 4, 7 and 9) are excluded from lipid rafts^11^. In non-neuronal cells, the soluble AC (sAC, AC10), are targeted to the subcellular compartments including mitochondria, the nucleus and the centrosome^51^,^52^. AC1 is involved in the regulation of ephrin-A-induced axonal retraction but does not modulate axon outgrowth^15^. All other transmembrane ACs are also unable to favour axonal elongation^35^. In contrast, sAC regulates intrinsic and netrin-1-stimulated axon outgrowth^35^,^53^ and is not involved in netrin-1-dependent axon guidance^54^. This is in agreement with our findings that define the AC1-containing lipid raft domains as a critical cAMP signalling compartment for axon guidance but not outgrowth. The latter may be regulated by sAC and cAMP signals located near the sites of sAC targeting^55^. The non-lipid raft fraction of the plasma membrane appears to constitute a third cAMP signalling compartment that has untill now no identified role in regulating the development of neuronal connectivity. Among the transmembrane ACs excluded from lipid rafts, AC2 and AC9 are expressed in developing RGCs^23^. The involvement of these AC subtypes in the development of retinal projections has not been directly assessed. Since the blockade of cAMP in the vicinity of the non-raft fraction of the plasma membrane does not affect the response of RGC axons to ephrin-As and the branching pattern in the SC, it seems unlikely that AC2 and AC9 contribute to the development of retinofugal projections.

In a wide range of cellular models, the control of local cAMP signals is dependent on specific subtypes of phosphodiesterases. In cell lines, phosphodiesterases contribute to local cAMP signalling in lipid rafts^12^. In cardiac myocytes and cortical pyramidal neurons, they are critical for independent activation of distinct cAMP-dependent pathways triggered by β1 or β2 adrenergic receptors^56^,^57^. The precise cellular targeting of phosphodiesterases relies on their binding to A kinase anchoring proteins (AKAPs)^58^. The lipid raft residency of a subset of AKAPs has been evaluated and AKAP79 has been found in this membrane subdomain^59^. Which phosphodiesterases and AKAPs are involved in RGC axon guidance and targeting remains to be investigated, and would provide a better understanding of the downstream pathways involved.

## Methods

### Animals

Pregnant C57BL6/J mice were purchased from Janvier. All animal procedures were performed in accordance with institutional guidelines (Université Pierre et Marie Curie, Comité Charles Darwin, Institut National de la Santé et de la Recherche Médicale). Animals were housed under 12 h light/12 h dark cycle. Embryos from dated mating (developmental stage stated in each section describing individual experiments) were not sexed during the experiments and the female over male ratio is expected to be close to 1.

### Chemicals and cell lines

Ephrin-A5 was purchased from R&D and dissolved in water at 1 mg ml^*−*1^ stock concentration and further diluted at a final concentration of 500 ng ml^*−*1^ in the recording medium. Forskolin (Sigma Aldrich) was dissolved in DMSO at 10 mM concentration and further diluted in the recording medium at 25 μM. IBMX (Sigma Aldrich) was dissolved in DMSO to a 100 mM concentration and further diluted at 100 μM in the recording medium.

HEK293 cells (from ATCC, not authenticated, not tested for mycoplasma contamination) were used to validate the plasma membrane localization of Lyn- and Kras-targeted constructs. Although this cell line is commonly misidentified, this did not affect the conclusion drawn using it since similar experiments were reproduced using retinal neurons.

### Plasmids

The non-targeted FRET sensor H147 (ref. 29), cAMP sponge-mCherry and its mutant that does not bind cAMP^30^ were used to generate the subcellularly restricted variants. All constructs used were subcloned into a pCX backbone^60^ and targeting to lipid rafts was achieved using a double-Lyn sequence (ATGGGCTGCATCAAGAGCAAGCGCAAGGACAAGATGGGCTGCATCAAGAGCAAGCGCAAGGACAAG) at the 5′ of the coding region of the cAMP sponge (using EcoRV-KpnI restriction sites), obtaining a Lyn-cAMP sponge. Targeting outside of lipid rafts was achieved using a Kras targeting sequence (AAGAAGAAGAAGAAGAAGAGCAAGACCAAGTGCGTGATCATG) at the 3′ end of the constructs. In the cAMP sponge-Kras, the mCherry tag was replaced by monomeric red fluorescent protein (mRFP), because of mCherry toxicity when combined with the Kras targeting sequence.

### Membrane fractionation by detergent-free method

Electroporated retinas were pelleted (195*g* for 5 min at 4 °C) and resuspended in 1.34 ml of 0.5 M sodium carbonate, pH 11.5, with protease inhibitor cocktail and phosphatase inhibitor cocktail 1, 2 and 3 (Sigma Aldrich). The homogenate was sheared through a 26-gauge needle and sonicated three times for 20 s bursts. The homogenate was adjusted to 40% sucrose by adding 2.06 ml of 60% sucrose in MES buffered saline (MBS) (25 mM MES, pH 6.4, 150 mM NaCl and 250 mM sodium carbonate), placed under a 5–30% discontinuous sucrose gradient, and centrifuged at 34,000 r.p.m. for 15–18 h at 4 °C in a Beckman SW-41Ti rotor. Nine fractions (1.24 ml each) were harvested from the top of the tube mixed with nine volumes of MBS, and centrifuged at 40,000 r.p.m. for 1 h at 4 °C (Beckman SW-41Ti rotor). Supernatants were discarded, and membrane pellets were resuspended in 100 μl of 1% SDS.

For immunoblotting, samples were separated on 4–15% Mini- Protean TGX Tris-Glycine-buffer SDS-PAGE (Biorad) and transferred onto 0.2 μm Trans-Blot Turbo nitrocellulose membranes (Biorad). Membranes were blocked for one hour at room temperature in 1 × TBS (10 mM Tris pH 8.0, 150 mM NaCl) supplemented with 5% (w/v) dried skim milk powder. Primary antibody incubation was carried out overnight at 4 °C, with the following antibodies: rabbit anti-GFP (1/200; A11122; Life Technologies; validated for this assay in Vitari *et al*.^61^), rabbit anti-DsRed (1/200; 632496; Clontech; validated for this assay in Hinson *et al*.^62^), rabbit anti-β-Adaptin (1/200; sc-10762; Santa Cruz; ; validated for this assay in Pagano *et al*.^63^) and rabbit anti-Caveolin (1/500; 610060; BD Transduction Laboratories; validated for this assay in Pagano *et al*.^63^). A goat anti-rabbit-HRP coupled secondary antibody was used for detection (Jackson ImmunoResearch, West Grove, PA, USA). After antibody incubations, membranes were extensively washed in TBS-T (TBS-containing 2.5% Tween-20). Western blots were visualized using the enhanced chemiluminescence method (ECL prime Western Blotting detection reagent, Amersham).

For lipid raft analysis (CtB staining), retinas were incubated at 37 °C for 1 h in serum free medium or serum free medium containing 1 U ml^*−*1^ cholesterol oxidase (Sigma Aldrich C8649) or 400 mU ml^*−*1^ SMase (Sigma Aldrich S8633). After membrane fractionation, 3 μl of each fraction, resuspended in MBS, were dot-blotted on nitrocellulose membrane, dried for 1 h and blocked for 1 h with 3% (w/v) dried skim milk powder at room temperature. The membrane was incubated overnight with HRP-conjugated cholera toxin β-subunit (Sigma Aldrich C3741) and detected with ECL Prime. All gels shown in the figures are provided uncropped in Supplementary Fig. 8.

### *Ex vivo* electroporation and culture of retinal explants

Mice were killed with CO_2_, and E13.5 embryos were isolated and kept in cold PBS. Embryos were decapitated and DNA was injected subretinally using an elongated borosilicate glass capillary (Harvard Apparatus). All constructs were co-electroporated with GFP subcloned in the same vector. The success of DNA injection was assessed using 0.03% Fast Green added to the DNA solution. The paddles of the electrode (CUY650P5, Sonidel) were placed at the bottom and at the top of the head, respectively^64^. Two poring pulses (square wave, 175 V, 5 ms duration, with 50 ms interval) followed by four transfer pulses (40 V, 50 ms and 950 ms interpulse) were applied. The protocol was repeated with inverted polarities. After electroporation, the retinas were isolated using a 21G needle and kept 24 h in culture medium (DMEM-F12 supplemented with 1mM glutamine (Sigma Aldrich), 1% penicillin/streptomicin (Sigma Aldrich), 0.01% BSA (Sigma Aldrich), 0.07% glucose), in a humidified incubator at 37 °C and 5% CO_2_. Non-electroporated retinas were isolated at E14.5. Retinas were cut into 200 μm squares and explants were plated on glass coverslips coated with 100 μg ml^*−*1^ poly-L-lysine (Sigma Aldrich) overnight at 37 °C and 20 μg ml^*−*1^ Laminin (Sigma Aldrich) for 3 h at 37 °C. Cells were cultured for 24 h in culture medium supplemented with 0.4% methyl cellulose and B27.

### Collapse assay

After 24 h of plating retinal explants, ephrin-A5 (500 ng μl^*−*1^)^15^ was added to the medium and the culture was fixed 1 h later, and immunolabeled with a mouse anti α-tubulin (1/1000; T6199; Sigma; validated for this assay in Nicol *et al*.^33^) and a phalloidin staining (Life Technologies). The retraction course was measured as the length of the trailing process, identified as the longest filopodia distal to the retraction bulb. SMase (400 mU ml^*−*1^)^63^ and cholesterol oxidase (2 U ml^*−*1^)^3^ were applied 90 and 120 min, respectively, before ephrin-A5 exposure and retinal explants were incubated at 37 °C.

### Retraction assay

Imaging was performed using an inverted DMI6000B Leica microscope, with a 40 × objective. Recording medium was replaced with a laminar flux. Temperature was kept at 37 °C during the whole recording. Retraction assays were performed by bathing the cells in a medium containing: 1 mM CaCl_2_, 0.3 μM MgCl_2_, 0.5 mM Na_2_HPO_4_, 0.5 mM NaH_2_PO_4_, 0.4 μM MgSO_4_, 4.25 mM KCl, 14 μM NaHCO_3_, 120 mM NaCl, 0.0004% CuSO_4_, 1.24 μM Fe (NO_3_)_3_, 1.5 μM FeSO_4_, 1.5 μM thymidine, 0.51 mM lipoic acid, 1.5 mM ZnSO_4_, 0.5 μM sodium pyruvate, 1 × MEM Amino Acids, 1 × non-essential amino acids, 25 mM Hepes, 0.5 mM putrescine, 0.01% BSA, 0.46% glucose, 1 mM glutamine, 2% penicillin streptomicin. Vitamin B12 and riboflavin were omitted because of their autofluorescence. DIC images were acquired using a CCD camera (ORCA-D2, Hamamatsu). Cells were imaged every 2 min for up to 90 min. Solution was perfused at a speed of 0.2 ml min^*−*1^. Cells were bathed in control medium for 30 min to measure the growth rate and eliminate immobile growth cones from the analysis. A single fluorescent image was acquired before acquisition of identified electroporated axons. Only axons that grew faster than 60 μm h^−1^ were included in the analysis. After 30 min, the culture medium was replaced by a medium containing ephrin-A5 (500 ng μl^*−*1^) and axons were imaged for 1 h. Axon trajectories were tracked using a manual tracking plugin for ImageJ (NIH) and the speed of axon outgrowth or retraction was computed.

### FRET imaging and data analysis

Images were taken every 30 s (every 20 s for Supplementary Fig. 4) for 30 min with the same experimental setup, using an oil immersion 63 × objective (1.40 NA). A CFP filter (436/20 nm) was used for excitation and emission was collected using the optical block of the dual CCD camera ORCA-D2 (CFP: 483/32 nm, YFP: 542/27 nm). Metamorph was used to collect images. Post-acquisition analysis was performed using ImageJ (NIH). Images were background corrected. CFP bleedthrough into the YFP channel was calculated in experiments using cells expressing only CFP. Experiments conducted with cells expressing only CFP measured a bleedthrough of 45%. Bleedthrough correction was computed as *F*_FRET_=*F*_YFP_−0.45 × *F*_CFP_. A clipping value excluding pixels with remaining background (outside the axon) was applied to images to compute the CFP over FRET ratio. The ratio of all pixels with grey value lower than the clipping value was set to 0. The ratio of the entire growth cone was averaged and normalized to the first value of the trace. Photobleaching was corrected based on the slope of the trace before the first stimulation.

### Optogenetics

Retinas were co-electroporated *ex vivo* with YFP and either Lyn-bPAC or bPAC-Kras. DIC images were acquired every 2 min for 30 min with a 40 × immersion objective. After monitoring growth for 30 min using a low intensity of transmitted light, axons were stimulated by shining one to five flashes of blue light (100 ms of light exposure per flash, 1 min between flashes). After stimulation, axons were monitored for an additional 60 min, using the low intensity of transmitted light. At the end of each experiment an image was acquired with the YFP and RFP filter to identify bPAC-expressing axons. The number of axons responding to light (either stopping or retracting) was counted and compared with non-expressing axons in the same dish to better compare axons in the same conditions. Stalling axons were identified as those stopping for more than 5 min, while axons were identified as retracting when retreating more than 5 μm during the 60 min following light stimulation.

### *In utero* electroporation

Pregnant wild-type females were anesthetized with isofluorane. Using a glass micropipette (Dutscher), the left eyes of E15.5 embryos were injected with a mix of two DNA constructs: Lyn-cAMP sponge (2.7 μg μl^*−*1^) and GFP (1.1 μg μl^*−*1^), cAMP sponge-Kras (1.8 μg μl^*−*1^) and GFP (1.1 μg μl^*−*1^), or Lyn-mutated cAMP sponge (2.7 μg μl^*−*1^) and GFP (1.1 μg μl^*−*1^). Retinas were electroporated with five pulses of 45 V for 50 ms every 950 ms (Nepagene electroporator). The positive electrode was placed on the injected eye and the negative electrode at the opposite side (CUY650P5, Sonidel)^64^,^65^. Sub-cutaneous injections of flunixin-meglumine (4 mg kg^*−*1^, Sigma) were applied for analgesia after the surgery. To increase the survival of the pups, a Swiss female mated one day earlier than the electroporated mice was used to nurse the pups. At P0, all but three Swiss pups were removed to stimulate nursing.

### Immunostaining

At P10, after anaesthesia with pentobarbital (545 mg kg^*−*1^), *in utero* electroporated pups were perfused transcardially with 4% paraformaldehyde (PFA) in 0.12 M phosphate buffer. Brains and eyes were postfixed overnight in 4% PFA. Whole- SC and electroporated retinas were dissected. The retinas were oriented with an incision in the peripheral ventral part. SC and retinas were washed in PBS, permeabilized in 1% Triton for 30 min, blocked in 0.1% Triton 10% horse serum in PBS for 1 h. The SC and retinas were incubated at 4 °C for 3 days in antibodies raised against DsRed (1/800, Clonetech California; validated for similar assays in Belle *et al*.^66^) and GFP (1/1,000, Aves Lab; validated for similar assays in Muzerelle *et al*.^67^) diluted in the blocking solution. Finally, the SC and retinas were washed in PBS three times for 10 min each, incubated at room temperature for 2 h in the secondary antibodies (Alexa 488 Donkey anti-Chicken, 1/200, Invitrogen; and CY3 Donkey anti-rabbit, 1/200, Jackson) diluted in the blocking solution and washed again in PBS 3 times for 10 min. SC and retinas were mounted in mowiol-Dabco. Three additional incisions were performed to flatten the retinas before mounting.

### Whole-mount SC imaging and analysis

Whole-mount SC were imaged using a Leica SP5 confocal and a × 20 objective. Since the signal-to-noise ratio was higher in the GFP channel, electroporated axons were imaged using GFP staining. Co-electroporation with an excess of GFP led most if not all the GFP-positive axons to express the cAMP sponge (Supplementary Fig. 2). Individual axons were manually reconstructed from the anterior SC and through the SC depth using Adobe Photoshop (without reconstructing the terminal arbor) (Supplementary Movie 3). Axons were qualitatively classified in three types: normal (with an elaborated terminal arbor and without ectopic branches along the axon), immature (without a terminal arbor and with ectopic branches along the axon) and abnormal (with an elaborated terminal arbor but with ectopic branches along the axon or remaining caudal branches).

### Statistics

No randomization method was used and data were not analyzed blind. Data were presented as mean values±standard error of the mean. Statistical significance was calculated using two-sided unpaired tests for non-parametric tendencies (Mann–Whitney or Kruskal–Wallis) or a *χ*^2^ test for axonal arbors comparison *in vivo* and the count bPAC-expressing axons stalling or retracting in response to light. This enables to avoid to test for distribution normality. Differences were considered statistically significant when *P*<0.05 (with additional Bonferroni adjustment for multiple comparisons). Statistical test data were as follows: Figure 1d, Mann–Whitney, *P*<0.0001, *U*=38287. Figure 5e comparison with GFP-expressing axons 60 min after ephrin-A5 application; Kruskal–Wallis *P*=0.0051, df=3, *χ*^2^=12.78; Dunn's *post-hoc* test, Lyn-cAMP sponge *P*=0.0011, mean rank difference=-28.01; cAMP sponge-Kras *P*>0.99, mean rank difference=−5.61; Lyn-mutated cAMP sponge *P*>0.99, mean rank difference=−7.96. Figure 6g, Kruskal–Wallis *P*=0.029, df=3, *χ*^2^=9.00; Dunn's *post-hoc* test, Lyn-bPAC *P*=0.0094, mean rank difference=14; bPAC-Kras *P*=0.50, mean rank difference=−2.5; Lyn-bPAC without light *P*>0.99, mean rank difference=−0.86. *Post-hoc* statistical power was computed using G*Power (*α*=0.05). Effect size and statistical power: Fig. 1d *d*=0.7770 and 1−*β*=1.0000; Fig. 5e comparison of Lyn-cAMP sponge- and GFP-expressing axons *d*=1.1837 and 1−*β*=0.9855. Figure 6g comparison of control and Lyn-bPAC-expressing axons *d*=0.9798 and 1−*β*=0.8544.

### Data availability

The authors declare that all data supporting the findings of this study are available within the article and its Supplementary information files or from the corresponding author upon reasonable request.

## Additional information

**How to cite this article:** Averaimo, S. *et al*. A plasma membrane microdomain compartmentalizes ephrin-generated cAMP signals to prune developing retinal axon arbors. *Nat. Commun.* 7:12896 doi: 10.1038/ncomms12896 (2016).

## Supplementary Material

Supplementary Information Supplementary Figures 1-8

Supplementary Movie 1 Axons of retinal ganglion cells retracted upon exposure to ephrin A5.

Supplementary Movie 2 The release of lipid raft-targeted bPAC light activation is sufficient to induce axon retraction.

Supplementary Movie 3 Reconstruction of individual retinal axon arbors in the Superior Colliculus.

## References

[b1] Luo L. & O'Leary D. D. M. Axon retraction and degeneration in development and disease. Annu. Rev. Neurosci. 28, 127–156 (2005). 16022592 10.1146/annurev.neuro.28.061604.135632

[b2] Guirland C., Suzuki S., Kojima M., Lu B. & Zheng J. Q. Lipid rafts mediate chemotropic guidance of nerve growth cones. Neuron 42, 51–62 (2004). 15066264 10.1016/s0896-6273(04)00157-6

[b3] Hérincs Z. . DCC association with lipid rafts is required for netrin-1-mediated axon guidance. J. Cell. Sci. 118, 1687–1692 (2005). 15811950 10.1242/jcs.02296

[b4] Lingwood D. & Simons K. Lipid rafts as a membrane-organizing principle. Science 327, 46–50 (2010). 20044567 10.1126/science.1174621

[b5] Sonnino S. & Prinetti A. Membrane domains and the ‘lipid raft' concept. Curr. Med. Chem. 20, 4–21 (2013). 23150999

[b6] Kamiguchi H. The region-specific activities of lipid rafts during axon growth and guidance. J. Neurochem. 98, 330–335 (2006). 16805828 10.1111/j.1471-4159.2006.03888.x

[b7] Bitsikas V., Riento K., Howe J. D., Barry N. P. & Nichols B. J. The role of flotillins in regulating Aβ production, investigated using Flotillin 1−/−, Flotillin 2−/− double knockout mice. PLoS ONE 9, e85217 (2014). 24465508 10.1371/journal.pone.0085217 PMC3897416

[b8] Le Lay S. & Kurzchalia T. V. Getting rid of caveolins: phenotypes of caveolin-deficient animals. Biochim. Biophys. Acta 1746, 322–333 (2005). 16019085 10.1016/j.bbamcr.2005.06.001

[b9] Ludwig A. . Flotillin microdomains interact with the cortical cytoskeleton to control uropod formation and neutrophil recruitment. J. Cell Biol. 191, 771–781 (2010). 21059848 10.1083/jcb.201005140 PMC2983060

[b10] Trushina E., Du Charme J., Parisi J. & McMurray C. T. Neurological abnormalities in caveolin-1 knock out mice. Behav. Brain Res. 172, 24–32 (2006). 16750274 10.1016/j.bbr.2006.04.024

[b11] Willoughby D. & Cooper D. M. F. Organization and Ca^2+^ regulation of adenylyl cyclases in cAMP microdomains. Physiol. Rev. 87, 965–1010 (2007). 17615394 10.1152/physrev.00049.2006

[b12] Agarwal S. R. . Role of membrane microdomains in compartmentation of cAMP signalling. PLoS ONE 9, e95835 (2014). 24752595 10.1371/journal.pone.0095835 PMC3994114

[b13] Depry C., Allen M. D. & Zhang J. Visualization of PKA activity in plasma membrane microdomains. Mol. Biosyst. 7, 52–58 (2011). 20838685 10.1039/c0mb00079e

[b14] Ming G. L. . cAMP-dependent growth cone guidance by netrin-1. Neuron 19, 1225–1235 (1997). 9427246 10.1016/s0896-6273(00)80414-6

[b15] Nicol X., Muzerelle A., Rio J. P., Métin C. & Gaspar P. Requirement of adenylate cyclase 1 for the ephrin-A5-dependent retraction of exuberant retinal axons. J. Neurosci. 26, 862–872 (2006). 16421306 10.1523/JNEUROSCI.3385-05.2006 PMC6675379

[b16] Song H. . Conversion of neuronal growth cone responses from repulsion to attraction by cyclic nucleotides. Science 281, 1515–1518 (1998). 9727979 10.1126/science.281.5382.1515

[b17] Edwards H. V., Christian F. & Baillie G. S. cAMP: novel concepts in compartmentalised signalling. Semin. Cell Dev. Biol. 23, 181–190 (2012). 21930230 10.1016/j.semcdb.2011.09.005

[b18] Nicol X., Hong K. P. & Spitzer N. C. Spatial and temporal second messenger codes for growth cone turning. Proc. Natl Acad. Sci. USA 108, 13776–13781 (2011). 21795610 10.1073/pnas.1100247108 PMC3158175

[b19] Shelly M. . Local and long-range reciprocal regulation of cAMP and cGMP in axon/dendrite formation. Science 327, 547–552 (2010). 20110498 10.1126/science.1179735

[b20] Nicol X. & Gaspar P. Routes to cAMP: shaping neuronal connectivity with distinct adenylate cyclases. Eur. J. Neurosci. 39, 1742–1751 (2014). 24628976 10.1111/ejn.12543

[b21] Ravary A. . Adenylate cyclase 1 as a key actor in the refinement of retinal projection maps. J. Neurosci. 23, 2228–2238 (2003). 12657682 10.1523/JNEUROSCI.23-06-02228.2003 PMC6742000

[b22] Welker E. . Altered sensory processing in the somatosensory cortex of the mouse mutant barrelless. Science 271, 1864–1867 (1996). 8596955 10.1126/science.271.5257.1864

[b23] Nicol X. . Role of the calcium modulated cyclases in the development of the retinal projections. Eur. J. Neurosci. 24, 3401–3414 (2006). 17229090 10.1111/j.1460-9568.2006.05227.x

[b24] Masada N., Ciruela A., Macdougall D. A. & Cooper D. M. F. Distinct mechanisms of regulation by Ca^2+^/calmodulin of type 1 and 8 adenylyl cyclases support their different physiological roles. J. Biol. Chem. 284, 4451–4463 (2009). 19029295 10.1074/jbc.M807359200 PMC2640985

[b25] Frisén J. . Ephrin-A5 (AL-1/RAGS) is essential for proper retinal axon guidance and topographic mapping in the mammalian visual system. Neuron 20, 235–243 (1998). 9491985 10.1016/s0896-6273(00)80452-3

[b26] Yanagisawa M., Ariga T. & Yu R. K. Cholera toxin B subunit binding does not correlate with GM1 expression: a study using mouse embryonic neural precursor cells. Glycobiology 16, 19G–22G (2006). 10.1093/glycob/cwl003 16964630

[b27] Wands A. M. . Fucosylation and protein glycosylation create functional receptors for cholera toxin. Elife 4, e09545 (2015). 26512888 10.7554/eLife.09545 PMC4686427

[b28] Neufeld E. B. . Intracellular trafficking of cholesterol monitored with a cyclodextrin. J. Biol. Chem. 271, 21604–21613 (1996). 8702948 10.1074/jbc.271.35.21604

[b29] Polito M. . The NO/cGMP pathway inhibits transient cAMP signals through the activation of PDE2 in striatal neurons. Front. Cell. Neurosci. 7, 211 (2013). 24302895 10.3389/fncel.2013.00211 PMC3831346

[b30] Lefkimmiatis K., Moyer M. P., Curci S. & Hofer A. M. ‘cAMP sponge': a buffer for cyclic adenosine 3′, 5′-monophosphate. PLoS ONE 4, e7649 (2009). 19888343 10.1371/journal.pone.0007649 PMC2766031

[b31] Stierl M. . Light modulation of cellular cAMP by a small bacterial photoactivated adenylyl cyclase, bPAC, of the soil bacterium *Beggiatoa*. J. Biol. Chem. 286, 1181–1188 (2011). 21030594 10.1074/jbc.M110.185496 PMC3020725

[b32] Schröder-Lang S. . Fast manipulation of cellular cAMP level by light *in vivo*. Nat. Methods 4, 39–42 (2007). 17128267 10.1038/nmeth975

[b33] Nicol X. . cAMP oscillations and retinal activity are permissive for ephrin signalling during the establishment of the retinotopic map. Nat. Neurosci. 10, 340–347 (2007). 17259982 10.1038/nn1842

[b34] Dhande O. S. . Role of adenylate cyclase 1 in retinofugal map development. J. Comp. Neurol. 520, 1562–1583 (2012). 22102330 10.1002/cne.23000 PMC3563095

[b35] Corredor R. G. . Soluble adenylyl cyclase activity is necessary for retinal ganglion cell survival and axon growth. J. Neurosci. 32, 7734–7744 (2012). 22649251 10.1523/JNEUROSCI.5288-11.2012 PMC3372574

[b36] Huai J. & Drescher U. An ephrin-A-dependent signalling pathway controls integrin function and is linked to the tyrosine phosphorylation of a 120-kDa protein. J. Biol. Chem. 276, 6689–6694 (2001). 11053419 10.1074/jbc.M008127200

[b37] Davy A. . Compartmentalized signalling by GPI-anchored ephrin-A5 requires the Fyn tyrosine kinase to regulate cellular adhesion. Genes Dev. 13, 3125–3135 (1999). 10601038 10.1101/gad.13.23.3125 PMC317175

[b38] Bocharov E. V. . Spatial structure and pH-dependent conformational diversity of dimeric transmembrane domain of the receptor tyrosine kinase EphA1. J. Biol. Chem. 283, 29385–29395 (2008). 18728013 10.1074/jbc.M803089200 PMC2662025

[b39] Stuermer C. A. O. Microdomain-forming proteins and the role of the reggies/flotillins during axon regeneration in zebrafish. Biochim. Biophys. Acta 1812, 415–422 (2011). 21147218 10.1016/j.bbadis.2010.12.004

[b40] Tassew N. G. . Modifying lipid rafts promotes regeneration and functional recovery. Cell Rep. 8, 1146–1159 (2014). 25127134 10.1016/j.celrep.2014.06.014

[b41] Pike L. J. Lipid rafts: heterogeneity on the high seas. Biochem. J. 378, 281–292 (2004). 14662007 10.1042/BJ20031672 PMC1223991

[b42] Song H. J., Ming G. L. & Poo M. M. cAMP-induced switching in turning direction of nerve growth cones. Nature 388, 275–279 (1997). 9230436 10.1038/40864

[b43] Murray A. J., Tucker S. J. & Shewan D. A. cAMP-dependent axon guidance is distinctly regulated by Epac and protein kinase A. J. Neurosci. 29, 15434–15444 (2009). 20007468 10.1523/JNEUROSCI.3071-09.2009 PMC6666109

[b44] Nishiyama M. . Cyclic AMP/GMP-dependent modulation of Ca^2+^ channels sets the polarity of nerve growth-cone turning. Nature 423, 990–995 (2003). 12827203 10.1038/nature01751

[b45] Averaimo S. & Nicol X. Intermingled cAMP, cGMP and calcium spatiotemporal dynamics in developing neuronal circuits. Front. Cell. Neurosci. 8, 376 (2014). 25431549 10.3389/fncel.2014.00376 PMC4230202

[b46] Zhou Z. . Photoactivated adenylyl cyclase (PAC) reveals novel mechanisms underlying cAMP-dependent axonal morphogenesis. Sci. Rep. 5, 19679 (2016). 26795422 10.1038/srep19679 PMC4726437

[b47] Woo S., Rowan D. J. & Gomez T. M. Retinotopic mapping requires focal adhesion kinase-mediated regulation of growth cone adhesion. J. Neurosci. 29, 13981–13991 (2009). 19890008 10.1523/JNEUROSCI.4028-09.2009 PMC2796108

[b48] Knöll B. & Drescher U. Src family kinases are involved in EphA receptor-mediated retinal axon guidance. J. Neurosci. 24, 6248–6257 (2004). 15254079 10.1523/JNEUROSCI.0985-04.2004 PMC6729544

[b49] Hausdorff W. P. . Tyrosine phosphorylation of G protein alpha subunits by pp60c-src. Proc. Natl Acad. Sci. USA 89, 5720–5724 (1992). 1378615 10.1073/pnas.89.13.5720 PMC49368

[b50] Wachten S. . Distinct pools of cAMP centre on different isoforms of adenylyl cyclase in pituitary-derived GH3B6 cells. J. Cell. Sci. 123, 95–106 (2010). 20016070 10.1242/jcs.058594 PMC2794711

[b51] Bundey R. A. & Insel P. A. Discrete intracellular signalling domains of soluble adenylyl cyclase: camps of cAMP? Sci. STKE 2004, pe19 (2004). 15126677 10.1126/stke.2312004pe19

[b52] Zippin J. H. . Compartmentalization of bicarbonate-sensitive adenylyl cyclase in distinct signalling microdomains. FASEB J. 17, 82–84 (2003). 12475901 10.1096/fj.02-0598fje

[b53] Wu K. Y. . Soluble adenylyl cyclase is required for netrin-1 signalling in nerve growth cones. Nat. Neurosci. 9, 1257–1264 (2006). 16964251 10.1038/nn1767 PMC3081654

[b54] Moore S. W. . Soluble adenylyl cyclase is not required for axon guidance to netrin-1. J. Neurosci. 28, 3920–3924 (2008). 18400890 10.1523/JNEUROSCI.0547-08.2008 PMC6670467

[b55] Stiles T. L., Kapiloff M. S. & Goldberg J. L. The role of soluble adenylyl cyclase in neurite outgrowth. Biochim. Biophys. Acta 1842, 2561–2568 (2014). 25064589 10.1016/j.bbadis.2014.07.012 PMC4262618

[b56] Mongillo M. . Fluorescence resonance energy transfer-based analysis of cAMP dynamics in live neonatal rat cardiac myocytes reveals distinct functions of compartmentalized phosphodiesterases. Circ. Res. 95, 67–75 (2004). 15178638 10.1161/01.RES.0000134629.84732.11

[b57] Castro L. R. V. . Type 4 phosphodiesterase plays different integrating roles in different cellular domains in pyramidal cortical neurons. J. Neurosci. 30, 6143–6151 (2010). 20427672 10.1523/JNEUROSCI.5851-09.2010 PMC6632585

[b58] Terrin A. . PKA and PDE4D3 anchoring to AKAP9 provides distinct regulation of cAMP signals at the centrosome. J. Cell Biol. 198, 607–621 (2012). 22908311 10.1083/jcb.201201059 PMC3514031

[b59] Delint-Ramirez I., Willoughby D., Hammond G. V. R., Ayling L. J. & Cooper D. M. F. Palmitoylation targets AKAP79 protein to lipid rafts and promotes its regulation of calcium-sensitive adenylyl cyclase type 8. J. Biol. Chem. 286, 32962–32975 (2011). 21771783 10.1074/jbc.M111.243899 PMC3190942

[b60] Okabe M., Ikawa M., Kominami K., Nakanishi T. & Nishimune Y. ‘Green mice' as a source of ubiquitous green cells. FEBS Lett. 407, 313–319 (1997). 9175875 10.1016/s0014-5793(97)00313-x

[b61] Vitari A. C. . COP1 is a tumour suppressor that causes degradation of ETS transcription factors. Nature 474, 403–406 (2011). 21572435 10.1038/nature10005

[b62] Hinson E. R. & Cresswell P. The antiviral protein, viperin, localizes to lipid droplets via its N-terminal amphipathic alpha-helix. Proc. Natl Acad. Sci. USA 106, 20452–20457 (2009). 19920176 10.1073/pnas.0911679106 PMC2778571

[b63] Pagano M. . Insights into the residence in lipid rafts of adenylyl cyclase AC8 and its regulation by capacitative calcium entry. Am. J. Physiol. Cell Physiol 296, C607–C619 (2009). 19158400 10.1152/ajpcell.00488.2008 PMC2660271

[b64] Petros T. J., Rebsam A. & Mason C. A. *In utero* and *ex vivo* electroporation for gene expression in mouse retinal ganglion cells. J. Vis. Exp. 31, e1333 (2009). 10.3791/1333 PMC3142865 19779401

[b65] Garcia-Frigola C., Carreres M. I., Vegar C. & Herrera E. Gene delivery into mouse retinal ganglion cells by *in utero* electroporation. BMC Dev. Biol. 7, 103 (2007). 17875204 10.1186/1471-213X-7-103 PMC2080638

[b66] Belle M. . A simple method for 3D analysis of immunolabeled axonal tracts in a transparent nervous system. Cell Rep. 9, 1191–1201 (2014). 25456121 10.1016/j.celrep.2014.10.037

[b67] Muzerelle A., Scotto-Lomassese S., Bernard J. F., Soiza-Reilly M. & Gaspar P. Conditional anterograde tracing reveals distinct targeting of individual serotonin cell groups (B5–B9) to the forebrain and brainstem. Brain Struct. Funct. 221, 535–561 (2014). 25403254 10.1007/s00429-014-0924-4 PMC4750555

