# Supplementary material for: A plasma membrane microdomain compartmentalizes ephrin-generated cAMP signals to prune developing retinal axon arbors
Source: Nat Commun. 2016 Oct 3;7:12896. doi: 10.1038/ncomms12896 (PMC5059439; doi:10.1038/ncomms12896)
Supplement: Supplementary Information — Supplementary Figures 1-8 [file ncomms12896-s1.pdf]

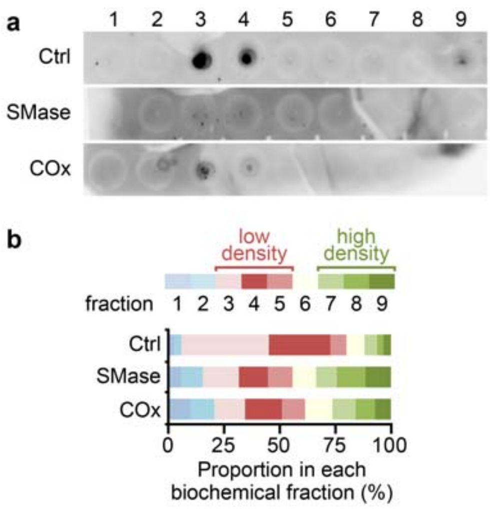

Supplementary Figure 1: SMase and COx perturb the enrichment of CtB in light density fractions. (a) CtB staining is enriched in fraction 3 and 4 (light density fractions) in control (ctrl) condition, but not in the presence of 2 different lipid raft disruptors, sphingomyelinase (SMase) or cholesterol oxidase (COx). **(b)** Proportion of CtB found in each biochemical fraction. The optical density (OD) of the dot in each fraction is quantified and normalized to the sum of the OD in all fractions. The proportion of the signal found in each fraction is shown. Each biochemical fraction is color-coded. Red tones code for the light density fractions (3 to 5), whereas green tones denote the high density fractions (7 to 9). From ≥ 3 experiments.

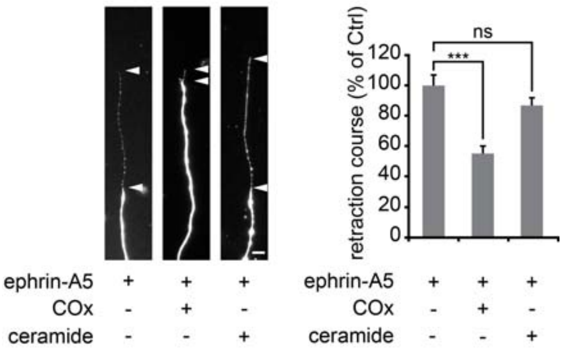

Supplementary Figure 2: Cholesterol oxidase (COx) but not ceramide reduces the ephrin-A5-induced retraction of retinal axons. Cultured RGC axons were treated with cholesterol oxidase or the SMase product ceramide prior to ephrin-A5. COx reduces the length of the trailing process of the retracting axons (encompassed by arrowheads). In contrast, the retracting filopodia is similar in untreated and ceramide-exposed axons. Scale bar, 10 µm; Data are mean ± SEM; Kruskal Wallis test; *** $P \leq 0.001$; ns, not significant.

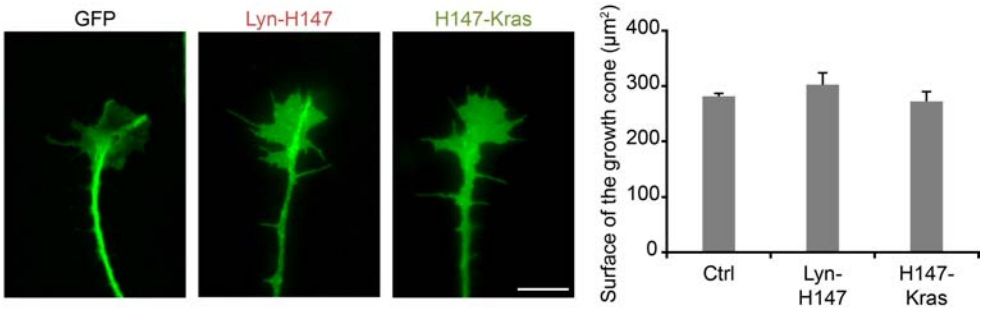

Supplementary Figure 3: Expression of Lyn-H147 or H147-Kras does not affect growth cone morphology. Growth cones expressing H147-Kras or Lyn-H147 are not distinguishable from those expressing GFP, with no detectable change in their morphology or area. Scale bar 10 µm. Data are mean ± SEM.

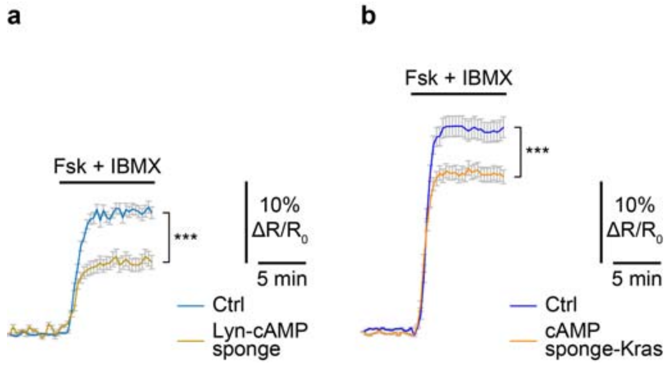

Supplementary Figure 4: Lipid-raft targeted and excluded cAMP sponge alter local cAMP signaling. (a) Retinal neurons expressing Lyn-H147 alone (blue trace) or in combination with Lyn-cAMP sponge (orange trace) were exposed to Fsk and IBMX to stimulate adenylyl cyclases and inactivate phosphodiesterases. This treatment generated a massive increase of cAMP that is reduced by Lyn-cAMP sponge expression. **(b)** Likewise H147-Kras was expressed alone (orange trace) or in combination with cAMP sponge-Kras (blue trace). Fsk and IBMX induced a large increase in cAMP that was attenuated when Lyn-cAMP sponge was co-expressed. Data are mean ± SEM. Mann Whitney test; *** P ≤ 0.001.

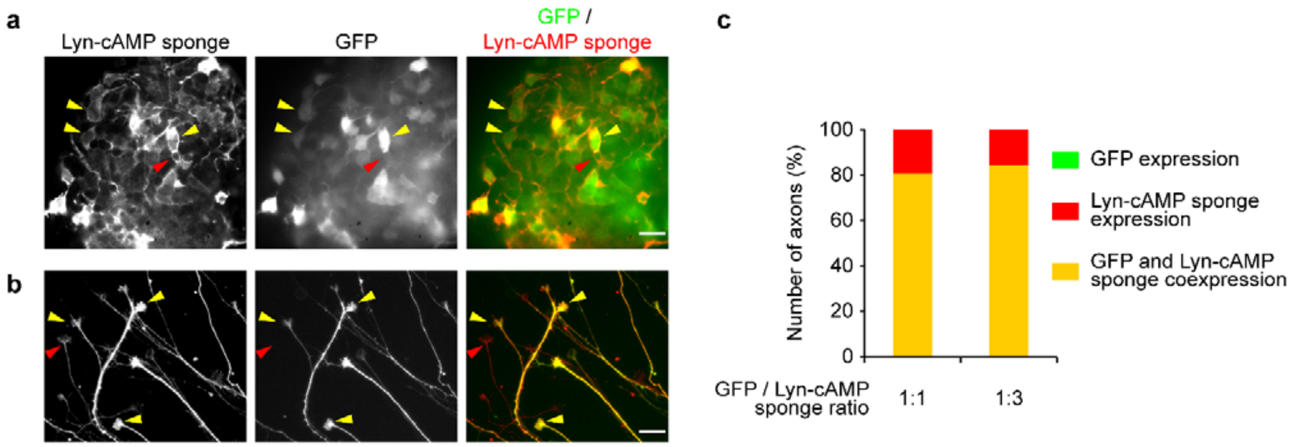

Supplementary Figure 5: Co-expression of GFP and Lyn-cAMP sponge in electroporated RGC axons. GFP and Lyn-cAMP sponge co-expression in **(a)** cell bodies and **(b)** axons of retinal explants co-electroporated with GFP or Lyn-cAMP sponge. All GFP-positive neurons and axons co-express Lyn-cAMP sponge (yellow arrowheads), whereas a few retinal cells and axons express Lyn-cAMP sponge alone (red arrowheads). **(c)** Quantification of GFP and Lyn-cAMP sponge co-expression in RGC axons electroporated with either 1:1 or 1:3 GFP/Lyn-cAMP sponge DNA ratio. With a 1:1 ratio, 81% of the electroporated axons express both GFP and Lyn sponge. With 1:3 ratio, 85% of the axons express both GFP and Lyn-cAMP sponge while 15% express only Lyn-cAMP sponge. No neuron or axon express only GFP. Scale bar 30 µm.

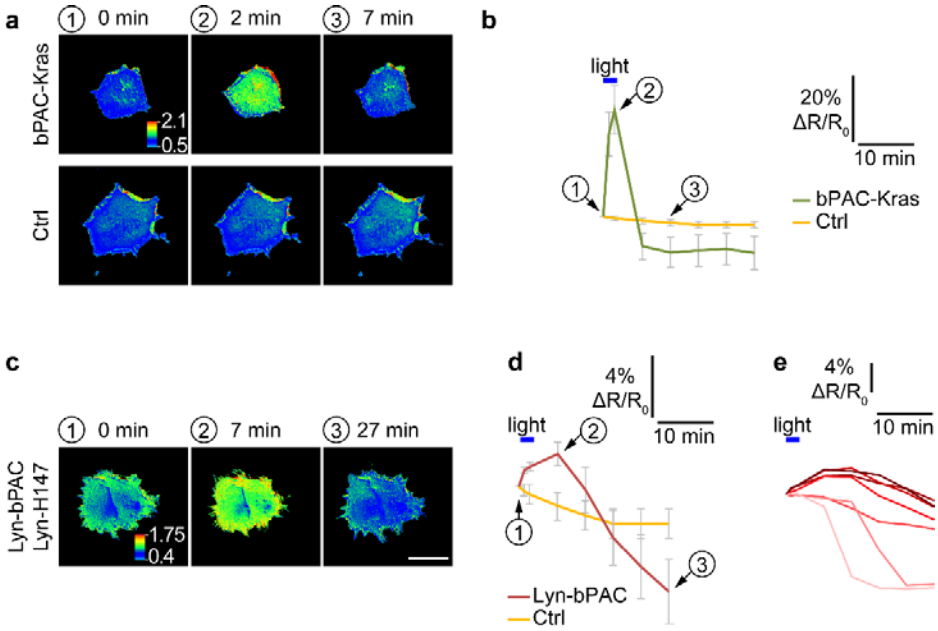

Supplementary Figure 6: Local modulation of cAMP concentration using subcellular targeting of bPAC. (a,b) HEK293 cells expressing the FRET sensor H147-Kras with or without bPAC-Kras are imaged. Images are acquired every minute for the first 3 time points (first imaging period). The wavelength exciting the FRET sensor overlaps with the activation spectrum of bPAC, enabling FRET imaging and light activation of bPAC with the same light flash. Cells are then imaged once every 5 minutes for 25 minutes (second imaging period). This reduction of the frequency of acquisition is sufficient to reduce bPAC stimulation and enable hydrolysis of bPAC-produced cAMP by phosphodiesterases. cAMP concentration increases in bPAC-expressing cells during the first imaging period (2) before decreasing during the second imaging period (3). In contrast, cAMP concentration is stable in cells expressing H147-Kras alone. **(c,d)** The same stimulation protocol is used for cells expressing the lipid raft-targeted Lyn-bPAC together with the lipid raft-targeted sensor (Lyn-H147). Lyn-bPAC induces an increase followed by a reduction of cAMP concentration. The response of individual cells is plotted in **(e)**. The delay before cAMP reduction is variable from cell to cell and may reflect different levels of activation of phosphodiesterases. Scale bar 20 µm. n ≥ 22 cells per condition. Data are mean ± SEM. Scale bar 10 µm.

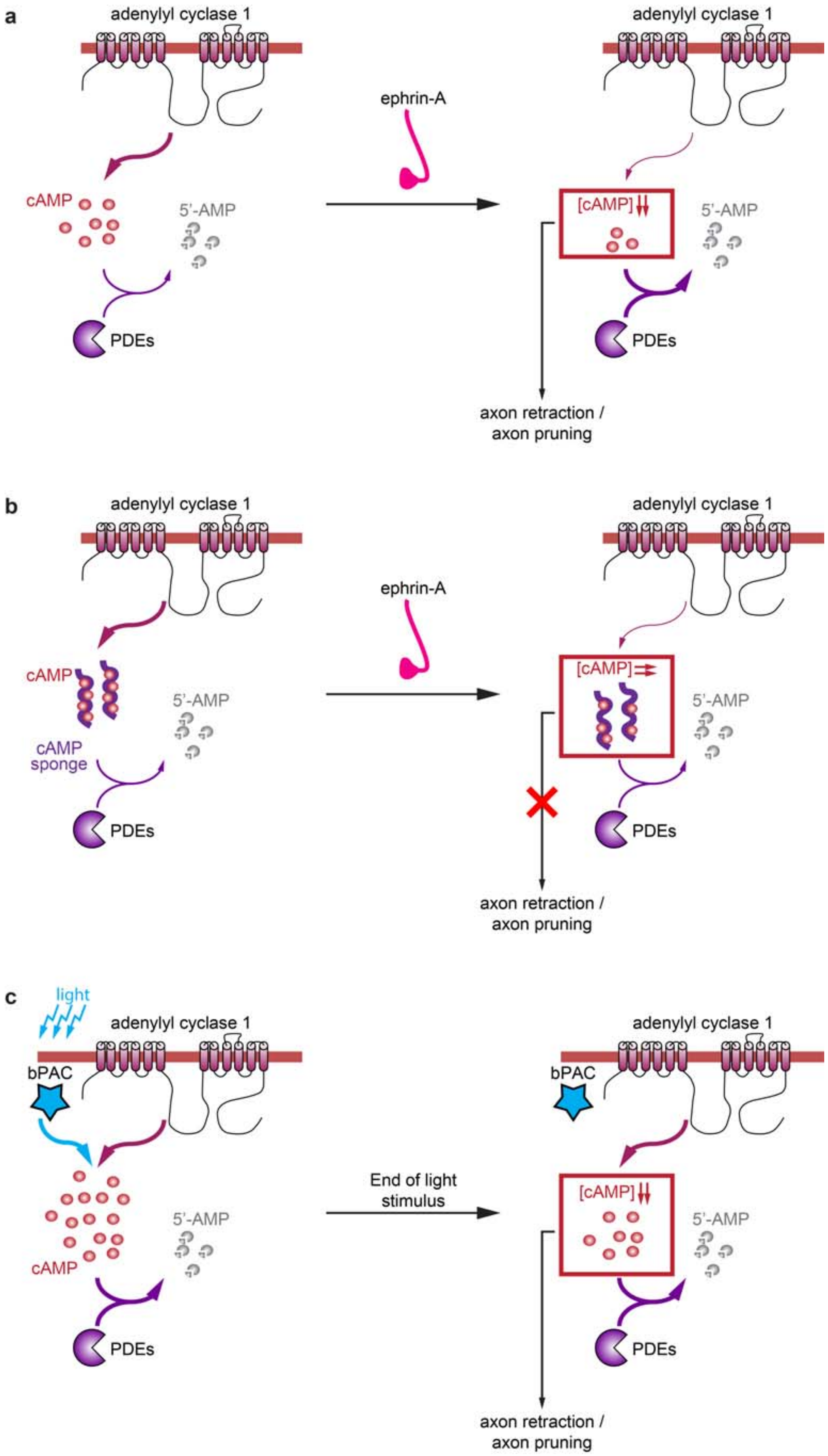
a
adenylyl cyclase 1
cAMP
5'-AMP
PDEs
ephrin-A
adenylyl cyclase 1
[cAMP]
5'-AMP
PDEs
axon retraction /
axon pruning
b
adenylyl cyclase 1
cAMP
5'-AMP
cAMP
sponge
PDEs
ephrin-A
adenylyl cyclase 1
[cAMP]
5'-AMP
PDEs
axon retraction /
axon pruning
c
light
adenylyl cyclase 1
bPAC
cAMP
5'-AMP
PDEs
End of light
stimulus
adenylyl cyclase 1
bPAC
[cAMP]
5'-AMP
PDEs
axon retraction /
axon pruning

Supplementary Figure 7: Model of dynamic modulation of cAMP concentration during ephrin-A-induced axon retraction. (a) Ephrin-A5 induces a reduction in cAMP concentration in growth cones, leading to axon retraction, likely through a modulation of the balance between adenylyl cyclase 1 and phosphodiesterases (PDEs) activity. **(b)** cAMP sponge expression reduces cAMP downstream signaling prior to ephrin-A5 application, reducing the resting concentration of available cAMP. This prevents a further decrease of cAMP concentration when growth cones are exposed to ephrin-A5, leading to the reduction of axon retraction *in vitro* and axon pruning *in vivo*. **(c)** Exposing bPAC expressing axons to light increases the concentration of cAMP without affecting axon outgrowth. After the end of the light stimulation, bPAC activity decreases and cAMP concentration decreases due to PDE activity. This drop of cAMP concentration leads to axon retraction.

Figure 1

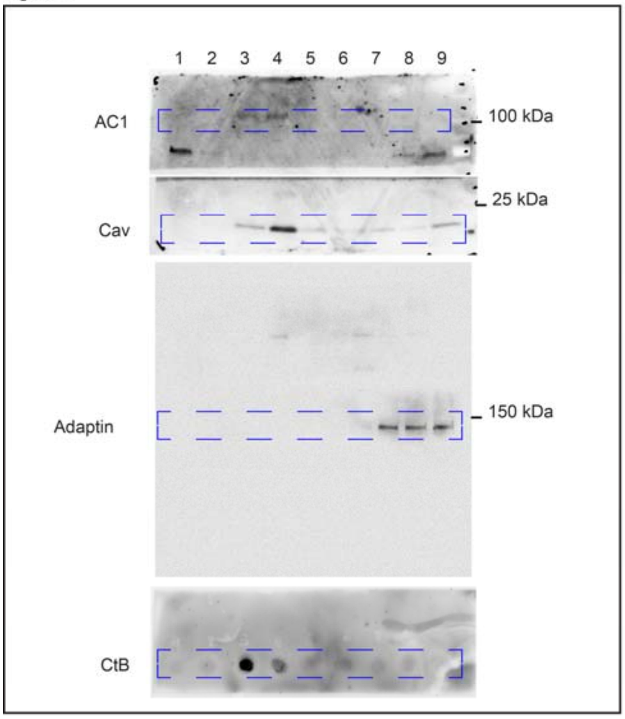

Figure 2

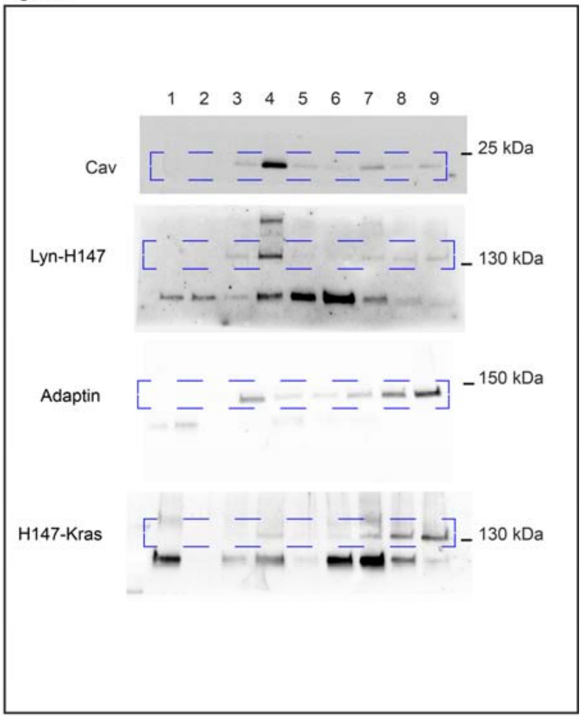

Figure 4

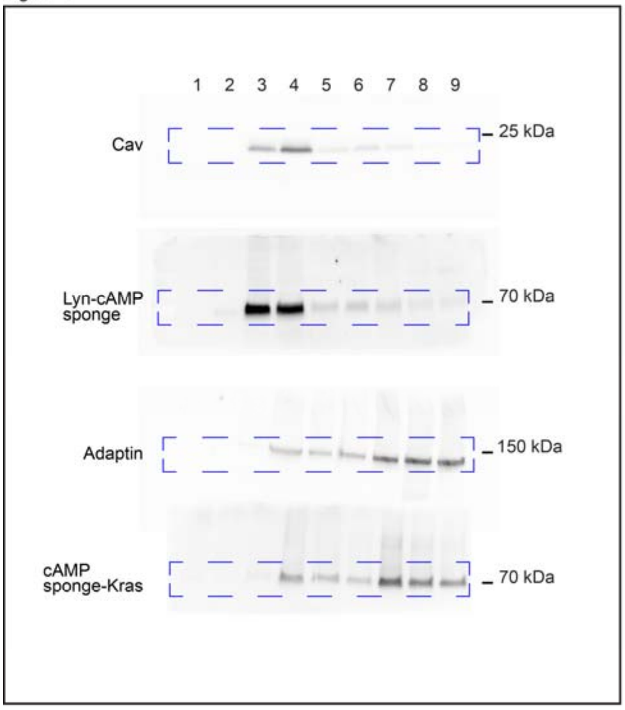

Figure 6

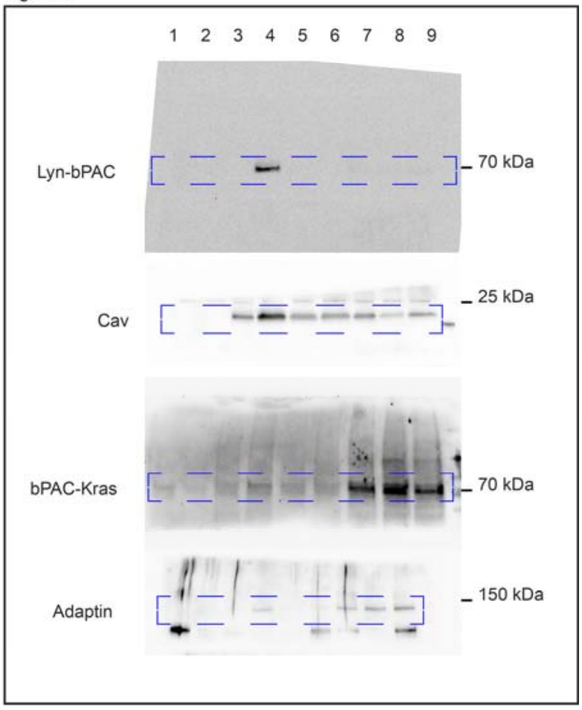

Supplementary Figure 8: Full pictures of the blots presented as cropped in the main text. Related to Fig1, 2, 4 and 6.
